# Nine Mitochondrial Genomes of Phasmatodea with Two Novel Mitochondrial Gene Rearrangements and Phylogeny

**DOI:** 10.3390/insects14050485

**Published:** 2023-05-22

**Authors:** Yani Yuan, Lihua Zhang, Ke Li, Yuehuan Hong, Kenneth B. Storey, Jiayong Zhang, Danna Yu

**Affiliations:** 1 College of Life Science, Zhejiang Normal University, Jinhua 321004, China; 2 Taishun County Forestry Bureau, Wenzhou 325500, China; 3 Department of Biology, Carleton University, Ottawa, ON K1S 5B6, Canada; 4 Key Lab of Wildlife Biotechnology, Conservation and Utilization of Zhejiang Province, Zhejiang Normal University, Jinhua 321004, China

**Keywords:** Phasmatodea, mitogenomes, rearrangement, phylogenetic relationships, tandem repeat

## Abstract

**Simple Summary:**

Stick and leaf insects are herbivorous species widely distributed in tropical and subtropical areas, disguising themselves as leaves, twigs, or moss through morphology and behavior to avoid visually hunting predators. Currently, Phasmatodea present difficulties in taxonomy, and their phylogeny is unresolved. Mitochondria, as maternally inherited organelles, also contain evolutionary information. Compared to nuclear genes, mitogenomes have become a powerful marker for inferring phylogenetic relationships due to advantages including fast evolution rates, conserved structure, and easy amplification. With rapid advances in sequencing technology and assembly algorithms, mitogenomes can be sequenced in a very cost-effective way. As of March 2023, there are thirty-seven complete or nearly complete Phasmatodea mitogenomes listed in the NCBI. Considering the richness of Phasmatodea, additional study is warranted. In the present study, nine new mitogenomes were sequenced to examine gene rearrangements and phylogenetic relationships within the Phasmatodea.

**Abstract:**

The classification of stick and leaf insects (Order Phasmatodea) is flawed at various taxonomic ranks due to a lack of robust phylogenetic relationships and convergent morphological characteristics. In this study, we sequenced nine new mitogenomes that ranged from 15,011 bp to 17,761 bp in length. In the mitogenome of *Carausis* sp., we found a translocation of *trnR* and *trnA*, which can be explained by the tandem duplication/random loss (TDRL) model. In the *Stheneboea repudiosa* Brunner von Wattenwyl, 1907, a novel mitochondrial structure of 12S rRNA-CR1-*trnI*-CR2-*trnQ*-*trnM* was found for the first time in Phasmatodea. Due to the low homology of CR1 and CR2, we hypothesized that *trnI* was inverted through recombination and then translocated into the middle of the control region. Control region repeats were frequently detected in the newly sequenced mitogenomes. To explore phylogenetic relationships in Phasmatodea, mtPCGs from 56 Phasmatodean species (composed of 9 stick insects from this study, 31 GenBank data, and 16 data derived from transcriptome splicing) were used for Bayesian inference (BI), and maximum likelihood (ML) analyses. Both analyses supported the monophyly of Lonchodinae and Necrosciinae, but Lonchodidae was polyphyletic. Phasmatidae was monophyletic, and Clitumninae was paraphyletic. Phyllidae was located at the base of Neophasmatodea and formed a sister group with the remaining Neophasmatodea. Bacillidae and Pseudophasmatidae were recovered as a sister group. Heteroptergidae was monophyletic, and the Heteropteryginae sister to the clade (Obriminae + Dataminae) was supported by BI analysis and ML analysis.

## 1. Introduction

Stick and leaf insects (Order Phasmatodea) have extraordinary mimetic abilities that are famous among the Insecta [1]. Currently, approximately 3400 species of Phasmatodea have been described worldwide, in 14 families and more than 500 genera, a moderately diverse insect group [2]. Since stick and leaf insects disguise themselves by mimicking plants, convergent evolution of related morphological characteristics and sexual dimorphism limits morphology-based classifications [3].

To categorize Phasmatodea, current taxonomists have focused on molecular data to explore affinities between species [4,5,6], particularly mitochondrial sequences since these organelles maintain a set of genetic materials independent of the nucleus [7]. Insect mitogenomes have a conserved circular structure, consisting of thirteen protein-coding genes (PCGs), twenty-two transfer RNAs (tRNAs), two ribosomal RNAs (16S rRNA and 12S rRNA), and a control region (CR) [8]. Scholars have used mitogenomes for taxonomic studies as they are easily sequenced, assembled, and annotated. Fast evolutionary rates and conserved gene structure make mitogenomes useful for estimating phylogenetic relationships [8,9,10]. Furthermore, mitogenomes can be obtained in a very low-cost way using next-generation sequencing (NGS) technology [11,12,13]. Although thirty-seven mitogenomes from Phasamatodea have now been sequenced, there is still a need for further work to represent their species richness.

In addition to sequence data from mitogenomes, gene rearrangements are also considered to be powerful phylogenetic markers [14]. In general, most mitochondrial gene arrangements among insects are identical to the ancestral insect [8]. However, exceptions have been found in various orders. For example, Thysanoptera has a rapid rate of evolution of the mitogenome structure [15,16,17]. Mantodea also generally exhibit gene rearrangements [18,19,20,21]. Mitochondrial gene rearrangements have also been observed in Hymenoptera [22], Hemiptera [23,24], Orthoptera [25,26], and Psocoptera [27]. Although patterns of gene rearrangement can be highly diverse, the mechanisms of mitochondrial gene rearrangement are poorly understood. At present, many rearrangements can be explained by the duplication and nonrandom loss model [28], the slipped-strand mispairing model [29], the tandem duplication/random loss model (TDRL) [30], or by recombination [31]. The latter two models are widely used to explain gene duplication. However, gene rearrangements rarely occur in Phasmatodea. To date, mitochondrial gene rearrangements have been reported in only five species: *Megalophasma granulatum* Bi, 1995 (*trnR*-*trnA*) [32], *Micadina brachyptera* Liu & Cai, 1994 (*trnA*-*trnR*-*trnS1*) [33], *Nanhuaphasma hamicercum* Chen & He, 2002 and *Orthomeria smaragdinum* Redtenbacher, 1906 (*trnN*-*trnR*) [34], and *Ramulus hainanense* Chen & He, 2002 (12Sr RNA-CR1-*trnM*-*trnQ*-*trnI*-CR2-*trnI*-*trnQ*-*trnM*). Genome rearrangements may be an additional characteristic to define phylogenetic relationships; however, assessment of potential synapomorphies among the Phasmatodea requires more mitogenomes.

Phylogenetic relationships in Phasmatodea have been discussed since this group became an independent order. Based on the presence or absence of a triangular field at the apex of the tibiae, Günther [35] divided Phasmatodea into two suborders (“Anareolatae” and “Areolatae”); however, their monophyly was not proven. Within Phasmatodea, Timematidea has been repeatedly confirmed to be a sister group of the Euphasmatodea (all remaining Phasmatodea) [5,36,37,38,39]. The monophyly of Heteropterygidae has been demonstrated [40,41,42]; however, some studies that included a few samples of Heteropterygidae did not recover monophyly [3,34,43]. Phylogenetic relationships between the three subfamilies of Heteropterygidae remain unresolved, although three distinct phylogenetic relationships among subgroups have been supported. According to morphological data, Hennemann et al. [44] supported Dataminae as the sister group to the clade of (Obriminae + Heteropteryginae), which had also been supported by some molecular analyses [6,41]. Two topologies ((Dataminae + Obriminae) + Heteropteryginae) and (Obriminae + (Dataminae + Heteropteryginae)) are supported by various molecular data [5,37,40,45,46]. However, due to the limited sample sizes, the internal phylogenetic relationships of Heteropterygidae remain unresolved. Another highly controversial issue was the phylogenetic taxonomy of the Phylliidae among the Euphasmatodea. Phylliidae are commonly known as true leaf insects due to their marked leaf-like appearance [47]. Therefore, Crampton [48] suggested the establishment of an order (Phyllioptera) to separate these from all other stick insects. However, Phylliidae was found to be a subordinate taxon within Euphasmatodea in several studies [40,49,50]. Recently, Bank et al. [51] recovered Phylliidae as a sister group to the remaining Neophasmatodea (all Euphasmatodea excluding Aschiphasmatidae), as was found by other studies [5,6,34,41,51]. However, Forni et al. [52], in an analysis of data derived from transcriptome splicing, found that Phylliidae had a close relationship to the Lonchodinae. This view has also been put forward in other studies [32,53,54]. Lonchodidae is a species-rich group with about 1229 species and 2 subfamilies (Lonchodinae and Necrosciinae), accounting for 36% of the total order [2]. Sister group relationships of these two subfamilies have not yet been determined. Morphologically, these two subfamilies both show long antennae and the absence of the area apicalis on the tibiae [55]. The sister group relationship between Lonchodinae and Necrosciinae is supported by multiple molecular analyses [5,42,56], but this needs further confirmation [52,53,54]. In addition, the monophyly of both subfamilies has been discussed repeatedly. Due to the heterogeneity of egg-capsule morphology and oviposition strategy, Sellick [57] considered the Necrosciinae to be polyphyletic. The monophyly of Necrosciinae was also supported by other studies [3,5,6]. Because misclassification often occurs in both subfamilies, addressing their monophyly requires a combination of morphology and molecular data. In the present study, nine mitogenomes of Phasmatodea were sequenced to examine mitochondrial gene rearrangement and phylogenetic relationships of Phasmatodea.

## 2. Materials and Methods

### 2.1. Sampling Collection

Stick insects were collected by netting and were stored in 100% alcohol at −20 °C. They were identified morphologically by Zhang. Detailed information about the samples was included in Table S1. All specimens were assigned a unique code, and corresponding vouchers were stored in Zhang Laboratory, College of Life Sciences, Zhejiang Normal University, Jinhua, China. 

### 2.2. DNA Extraction and Sequencing

Total DNA was extracted from the foreleg muscle using Ezup Column Animal Genomic DNA Purification Kit (Sangon Biotech Company, Shanghai, China) following manufacturer instructions. Two mitogenomes (*Lopaphus albopunctatus* Chen & He, 2004 and *Marmessoidea bispinus* Burmeister, 1838) were obtained by Sanger sequencing. Eight fragments of each mitogenome were successfully amplified using universal primers [58], and specific primers were designed in Primer Premier 5.0 [59] to fill gaps between fragments. PCR conditions for normal (amplified fragment < 3000 bp) and long (amplified fragment > 3000 bp) were as described in Zhang et al. [58]. Agarose gel electrophoresis (1% concentration) was used to estimate PCR product length. PCR products were bidirectionally sequenced by Sangon Biotech Company (Shanghai, China).

For the other seven species, DNA extract concentrations of more than 25 ug/mL were sent to BGI Tech Inc. (Shenzhen, China) for next-generation sequencing (NGS). The genomic DNA was sequenced using the Illumina HiSeq 2000 platform with 150 bp paired-end reads. The whole genome was randomly sheared and amplified by “bridge PCR”. After quality assessment of raw sequencing data with fastQC, clean data were used for genome assembly.

### 2.3. Mitogenome Assembly, Annotation and Sequence Analyses

Sanger sequencing was manually checked and assembled using SeqMan in the DNASTAR Package v.7.1 [60], and NGS were assembled in NOVOPlasty v.4.2 [61], GetOrganelle v.1.7.1 [62], and MitoZ [63], to ensure the consistency of the concatenated data. tRNA genes were identified using MITOS (http://mitos.bioinf.uni-leipzig.de/index.py, accessed on 15 August 2022) [64]. tRNAScan-SE (http://lowelab.ucsc.edu/tRNAscan-SE/index.htm, accessed on 24 December 2022) [65] and MITOS were used to predict tRNA secondary structures. The 13 PCGs were manually annotated using start codons (ATN, GTG, TTG) and stop codons (T, TA, TAA, TAG). The translation function of Mega 7.0 [66] was used to identify open reading frames with the invertebrate mitochondrial genetic code [67]. rRNA genes (12S rRNA and 16S rRNA) were identified by alignment with homologous genes from other sticks insects with ClustalW in Mega 7.0 [66]. Tandem repeats in the control region were detected by Tandem Repeat Finder v 4.09 (https://tandem.bu.edu/trf/home, accessed on 30 December 2022) [68]. PhyloSuite v.1.2.2 [69] was used to calculate AT content, codon usage, and relative synonymous codon usage (RSCU) for PCGs. Mitogenomes maps were drawn in CG View (http://cgview.ca/, accessed on 28 December 2022) [70]. GC- and AT-skews were calculated according to the formulas: AT-skew = (A − T)/(A + T), GC-skew = (G − C)/(G + C) [71].

### 2.4. Phylogenetic Analyses

A total of 56 stick insects of mitogenomes were used to explore their phylogenetic relationships within the Phasmatodea. These included the 9 new mitogenomes sequenced in this study and 47 mitogenomes from the NCBI [32,33,34,39,45,52,53,54,72,73]. Two species from Grylloblattodea and Mantophasmatodea were used as outgroups to root the phylogenetic tree (Table S1) [73]. Analysis of the saturation of each codon with DAMBE 7.3.11 showed that the values of Iss were less than Issc, indicating that no codon position used was saturated (Table S2) [74]. All 13 PCGs of the mitochondrial genes were used to build BI and ML phylogenetic trees. Each mitochondrial PCG was separately aligned with MAFFT v 7.475 [75]. Gblocks 0.91b [76] was used to remove poorly aligned regions. PCGs were concatenated in PhyloSuite v 1.2.2 [69] to form the nucleotide dataset. PartionFinder 2.2.1 [77] was used to optimize partition substitution models (Table 1). The BI tree was reconstructed in MrBayes 3.2 [78]. This involved 1000 million generations with sampling every million generations and the first 25% burn-in discarded. ML analysis was performed in IQ-TREE v.2.1.2 [79] with 1000 bootstrap replicates. FigTree v.1.4 was used to visualize evolutionary trees [80].

## 3. Results

### 3.1. Basic Features of Mitogenomes and Gene Rearrangement

**Figure 1:**
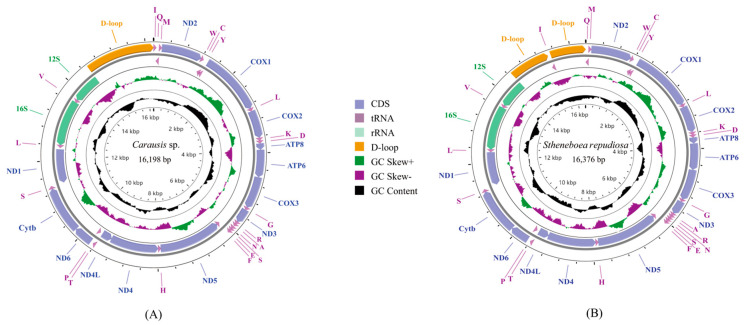
Complete mitogenome maps of (**A**) *Carausis* sp. and (**B**) *Stheneboea repudiosa*. Mitochondrial genes located in the outer circle are encoded by the J-strand, and those located in the inner circle are encoded by the N-strand. The different kinds of tRNA are represented by amino acid abbreviations. GC content and GC-skew are plotted as the deviation from the average value of the entire sequence.

**Figure 2:**
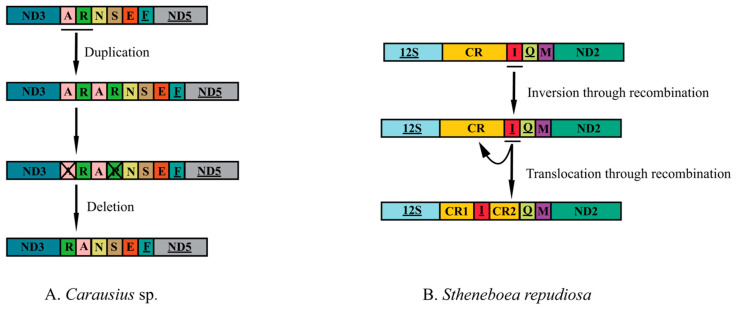
Mitochondrial gene arrangement and the putative rearrangement mechanism in (**A**) *Carausis* sp. and (**B**) *Stheneboea repudiosa*. Different genes are shown in different box colors. The order of the remaining mitogens is the same as the ancestral insect. Underlined genes are encoded by the N-strand, and those without underlines are encoded by the J-strand. The symbol ‘×’ represents the loss of the gene.

Seven complete mitogenomes and two nearly complete mitogenomes were sequenced. All genomes were deposited in GenBank. The control region of *L. albopunctatus* was not amplified, and the control region of *Pulchriphyllium giganteum* Hausleithner, 1984 was incompletely assembled. The 9 newly sequenced mitochondrial genomes ranged from 15,011 to 17,761 base pairs (bp) (Figure 1 and Figure S1). Differences in lengths were mainly due to the variation in control region size, genetic overlapping regions, and intergenic spacers. Overlap between genes ranged from 1 to 8 bp (Table S3). All the newly sequenced mitogenomes had the same 8 bp (AAGCCTTA) overlap between the *trnW* and *trnC* genes and the same 4 bp (ATAA) overlap between the ATP8 and ATP6 genes. Intergenic regions generally ranged from 1 to 19 bp (Table S3); however, *Pulchriphyllium bioculatum* Gray, 1832 had a 198 bp non-coding region (NCR) between *trnS2* and ND1. Each mitogenome had a high A + T content ranging from 73.8% (*P. giganteum*) to 79.3% (*L. albopunctatus*) (Table S4). All mitogenomes showed a positive AT-skew ranging from 0.160 (*Carausius* sp.) to 0.236 (*P. giganteum*) and negative GC-skew from −0.255 (*P. bioculatum*) to −0.118 (*L. albopunctatus*). Gene order of the newly sequenced mitogenomes was the same as the ancestral insects, except for *S. repudiosa* and *Carausius* sp. (Figure 2). In *S. repudiosa*, *trnI* was inverted and translocated to within the control region, resulting in the order CR1-*trnI*-CR2-*trnQ*-*trnM*. In *Carausius* sp., the gene block *trnA*-*trnR* was rearranged to *trnR*-*trnA*.

### 3.2. Protein-Coding Genes and Codon Usages

The longest PCG was COX1 at 1534 bp (Table S5). Nine PCGs were located on the J-strand (ATP6, ATP8, COX1, COX2, COX3, Cytb, ND2, ND3, ND6) and had a positive AT-skew and negative GC-skew, Four PCGs located on the N-strand (ND1, ND4, ND4L, ND5) showed the opposite (Table S5). Codon usage of PCGs is shown in Table S6 and Figure S2. The most frequently used codon in each species was AUA (Met), except for *L. albopunctatus*, which mostly used UUA (Leu). Relative synonymous codon usage (RSCU) was the highest for UUA (Leu). Amino acid usage (Table S7) showed that Met, Phe, Ile, and Leu were the most common amino acids, with Met accounting for more than 10% of the total amino acids. Cys and Arg were the least frequently used amino acids. The start and stop codons (Table S8) showed that ATN (N representing A, T, C, or G) was used as the start codon in almost all PCGs. TTG was start codon only in ND1 in two species (*Carausius* sp., *S. repudiosa*). TAA was the most common stop codon. Except for *S. repudiosa*, the incomplete stop codon T was used in COX2 and COX1 of all the newly sequenced mitogenomes.

### 3.3. Transfer RNA and Ribosomal RNA Genes

The total length of the 22 tRNAs ranged from 1425 bp to 1485 bp. All tRNAs were in the same position as in the ancestral insects, except for *S. repudiosa*, which had its *trnI* located on the N-strand (Table S2). The majority of tRNAs could be folded into canonical cloverleaf structures (Figure S3). Except for *Sungaya inexpectata* Zompro, 1996 and *Lopaphus sphalerus* Redtenbacher, 1908, the loss of the dihydrouridine (DHU) arm occurred in *trnS1* of all species. 

The length of 16S rRNA ranged from 1263 bp to 1305 bp and that of 12S rRNA ranged from 760 bp to 790 bp (Table S2). Both rRNAs showed a negative AT-skew, a positive GC-skew, and high AT content (from 76.3% to 80.7%) (Table S3). 

### 3.4. Control Region

For the seven complete mitogenomes reported in this study, the length of the control regions ranged from 1660 bp (*Carausius* sp.) to 3107 bp (*L. sphaleru*) (Table S3). The control regions of *S. repudiosa* were divided into two parts: 980 bp (CR1) and 862 bp (CR2). Control regions of *P. giganteum* and *L. albopunctatus* mitogenomes were incompletely sequenced, with lengths of 2147 bp and 386 bp, respectively. Complete control regions had high A + T content ranging from 71.4% to 82.1%.

**Figure 3:**
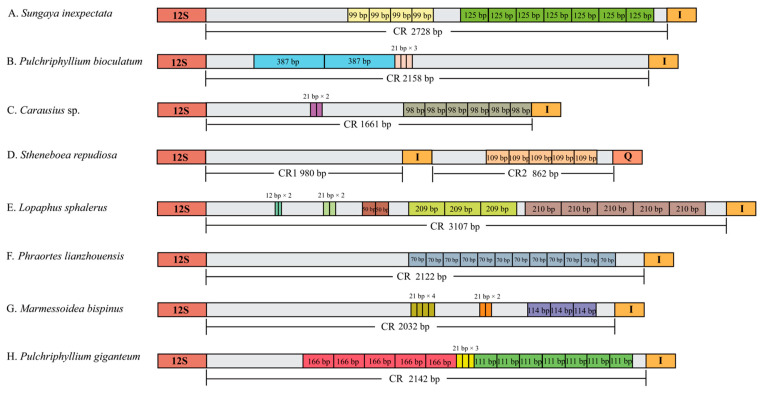
Schematic diagram of tandem repeat arrangements in the CR of eight stick insect species (**A**–**H**). Species names are annotated on the left side of the figure. Extent and length of the control regions are marked below the bar. Different tandem repeat sequences are represented by different colored boxes, with the length of each tandem repeat fragment annotated inside the box. 12S: 12S rRNA; I: *tnrI*; Q: *trnQ*.

Except for the control region of *L. albopunctatus*, tandem repeat regions were detected in the control regions of the eight other species (Figure 3). There were numerous forms of tandem repeats. The *S. inexpectata* mitogenome contained 4 × 99 bp and 7 × 125 bp repeats. *P. bioculatum* had adjacent 2 × 387 bp and 3 × 21 bp repeats. *Carausius* sp. contained 6 × 98 bp repeats and 2 × 21 bp repeats. *S. repudiosa* contained 5 × 109 bp repeats in CR2. *L. sphalerus* contained 3 × 209 bp, 5 × 210 bp 2 × 12 bp, 2 × 21 bp, and 2 × 50 bp repeats, respectively. *Phraortes lianzhouensis* Chen & He, 2008 contained 12 × 70 bp repeat. *M. bispinus* had 3 × 114 bp, 4 × 21 bp, and 2 × 21 bp repeats. *P. giganteum* contained 5 × 166 bp, 3 × 21 bp, and 7 × 111 bp repeats.

### 3.5. Phylogenetic Analyses

**Figure 4:**
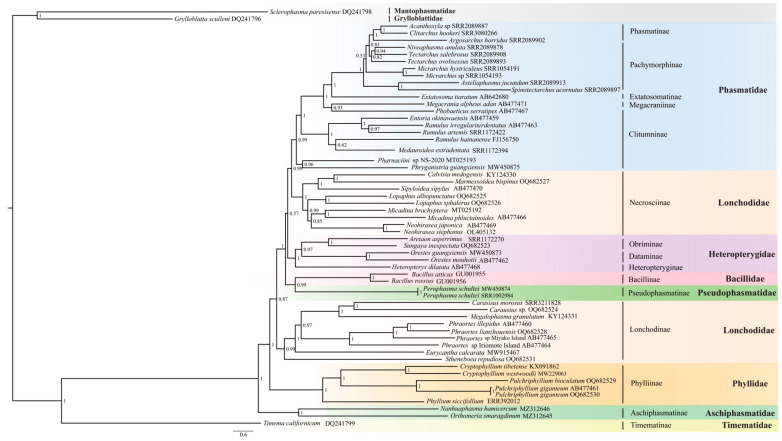
Phylogenetic relationships of Phasmatodea among 56 species inferred from BI analyses based on the nucleotide dataset of the 13 PCGs. Two species from Grylloblattodea and Mantophasmatodea were chosen as the outgroup. Posterior probabilities (PP) of BI analyses are shown in the nodes. GenBank or sequence read archive (SRA) numbers are annotated after the species name. Different families are highlighted by different colors.

**Figure 5:**
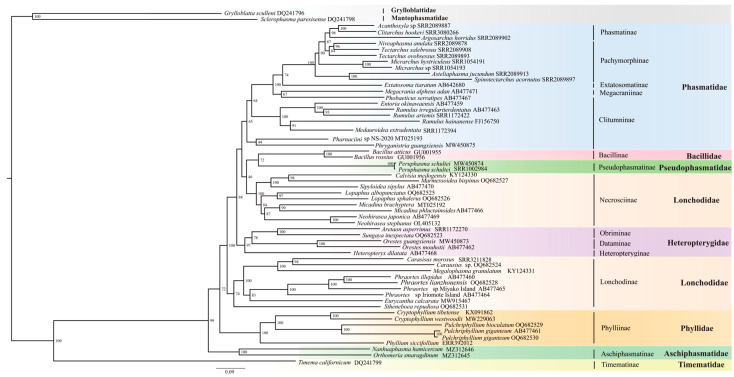
Phylogenetic relationships of Phasmatodea among 56 species inferred from ML analyses based on the nucleotide dataset of the 13 PCGs. Two species from Grylloblattodea and Mantophasmatodea were chosen as the outgroup. Bootstrap values (BP) of the ML analyses are shown at the nodes. GenBank or sequence read archive (SRA) numbers are annotated after the species name. Different families are highlighted by different colors.

In this study, BI and ML trees (Figure 4 and Figure 5) were constructed based on the concatenated 13 PCGs of the mitogenomes, with the GTR + I + G model. BI and ML trees were almost identical, the main difference being the position of (Bacillidae + Pseudophasmatidae). In the ML phylogenetic tree, (Bacillidae + Pseudophasmatidae) is sister to Necrosciinae, whereas the BI analysis revealed their distant phylogenetic relationship and Phasmatidae sister to Necrosciinae. Both BI and ML trees recovered the following results: (ⅰ) Timematidae was sister to all remaining Phasmatodea (=Euphasmatodea); (ⅱ) Aschiphasmatidae was sister to the remaining Euphasmatodeans (=Neophasmatodea); (ⅲ) Phylliidae was sister to remaining Neophasmatodea; (ⅳ) Lonchodidae was a polyphyletic group and its two subfamilies (Lonchodinae and Necrosciinae) clustered on different branches, both Lonchodinae and Necrosciinae were monophyletic; (ⅴ) Bacillidae and Pseudophasmatidae were sister groups; (ⅵ) Phasmatidae was rendered as a monophyletic group. At the subfamily level, Phasmatinae was monophyletic, whereas Clitumninae and Pachymorphinae were paraphyletic; (ⅶ) The monophyly of Heteroptergidae, and Heteropteryginae was sister to the clade (Obriminae + Dataminae).

## 4. Discussion

### 4.1. Non-Coding Regions and Control Regions of Mitogenomes

Published mitogenomes of Phasmatodea have genic intervals of 36 bp (*Phraortes* sp. 1 NS-2020, MT025191) to 83 bp (*Pharnaciini* sp. NS-2020, MT025193) [33]. Here, a 198 bp NCR between *trnS2* and ND1 was found in *P. bioculatum*, the longest gene interval yet detected in Phasmatodea mitogenomes. Tandem repeats were not detected in this 198 bp NCR using Tandem Repeat Finder v 4.09 [68]. The NCR matched part of ND1 with a similarity of 73.14%, which is likely a part of the ND1 gene that was lost randomly after its duplication. The TDRL mechanism has been widely used previously to explain the existence of mitochondrial NCRs [81]. 

On account of the inconsistency in the number of copies (about 2~50 copies) and the length of CR tandem repeats (about 8~700 bp), the length of the control region is highly variable [82,83]. In Phasmatodea, the control region length varied from 774 bp (*Ramulus hainanense* FJ156750) to 3429 bp (*Neohirasea stephanus* Redtenbacher, 1908 OL405132). Full sequencing of CRs is challenging due to the high A + T content and the large number of tandem repeats [84]. Tandem repeats were detected in the eight newly sequenced mitogenomes and were frequently found in the 3′ end of the control region in Phasmatodea [33,45,72]. Slipped strand mismatches during mtDNA replication are thought to be responsible for the formation of CR repeats [29]. Concerted evolution may be responsible for the small sequence differences observed between the same type of repeat units, but the specific mechanism is not yet known [85].

### 4.2. Gene Rearrangement

Although mtgene order differs between major animal lineages, it tends to be highly preserved within lineages, and gene rearrangements are assumed to be special [86]. In the present study, two mitochondrial gene rearrangements in Phasmatodea were described (Figure 2). One gene rearrangement was found in the ARNS1EF gene cluster. The gene block *trnA*-*trnR* was rearranged to *trnR*-*trnA* in *Carausius* sp. and was shared with *M. granulatum* [32]. This rearrangement is found in other species, including *Camarochiloides weiweii* Chen, Liu, Li & Cai, 2019 (Hemiptera: Pachynomidae) [23], *Anopheles peditaeniatus* Leicester, 1908, and *Anopheles nitidus* Harrison, Scanlon & Reid, 1973 (Diptera: Culicidae; Anophelinae) [87]. This rearrangement can be explained by the tandem duplication–random loss (TDRL) model [28]: first, *trnA*-*trnR* was duplicated to *trnA*-*trnR*-*trnA*-*trnR,* and subsequently, the first *trnA* and the second *trnR* were lost, generating the rearrangement *trnR*-*trnA*. 

Another rearrangement of gene structures also appeared in *S. repudiosa*, which showed the following rearrangement 12S rRNA-CR1-*trnI*-CR2-*trnQ*-*trnM*. This has not previously been reported in Phasmatodea. The low homology between CR1 and CR2 suggests that the TDRL model was unable to account for this as it cannot generate inversions [31,88]. Inversion and translocation of mitochondrial genes are found in several orders [19,89,90], especially in Hymenoptera [91,92]. Inversion and translocation of *trnI* and *trnM* formed the rearrangement structure (12S rRNA-*trnI*-CR1-*trnM*-CR2-*trnQ*) in *Aphidius gifuensis* Ashmead, 1906 (Hymenoptera: Ichneumonoidea: Braconidae) [93]. In addition, inversion and translocation occur widely between the ND3-ND5 genes of Hymenoptera [94]. Based on the above rearrangement pattern, we propose that the structure of 12S rRNA-CR1-*trnI*-CR2-*trnQ*-*trnM* can be explained by internal inversion of *trnI*, followed by its translocation to the middle of the control region. Recombination is considered to be the possible mechanism of gene inversion and translocation [95,96]. Gene rearrangement could be a powerful tool for inferring phylogeny and has been applied to the classification of some taxa [16,18,97]. To date, in Phasmatodea, rearrangement of *trnN*-*trnR* has only been reported in Aschiphasmatidae, and *trnR*-*trnA* has only been found in Lonchodidae. More types of rearrangements in Phasmatodea may yet remain to be discovered and studied.

### 4.3. Phylogenetic Relationship within Phasmatodea

Large-scale phylogenetic analysis of Phasmatodea has used both mitochondrial and nuclear genes [5,36,42]; however, phylogenetic analysis of mitogenomes has involved a limited number of species, suggesting the need for further sequencing of mitogenomes in this taxon. [34,45,98]. The phylogenetic status of Timematidae and Aschiphasmatidae tended to be stable, whether based on mitogenomes [32,33,34] or molecular markers [36,41,51,56]. In our analysis, Pseudophasmatidae was sister to Bacillidae. Bradler et al. [56] used nuclear genes (18S, 28S, H3) and mitochondrial genes (cox1, cox2, 12S, 16S) also recovered this conclusion. In recent studies, Pseudophasmatidae was sister to Agathemeridae [36,41,51], which was also supported by transcriptomic data [37,38]. The whole mitochondrial genome of the family Agathemeridae has not yet been reported, so this family could not be included in our systematic analysis. Due to its unique leaf-like appearance, Phyllidae is easily distinguishable from other stick insects. Our analysis showed that Phyllidae diverged from the remaining Neophasmatodea, which is consistent with many previous studies [5,6,34,41,51]. The sister relationship of Phyllidae with Lonchodinae was also recovered in molecular studies, but with low bootstrap support [52,54]. Monophyletic Necrosciinae and Lonchodinae constitute the group Lonchodidae, which has many contradictory phylogenetic relationships. In past years, Necrosciinae was classified within Diapheromeridae, and Lonchodidae was classified in Phasmatidae [1]. However, Robertson et al. [5] formally elucidated in their article that classify Necrosciinae and Lonchodinae into Lonchodidae. A sister relationship of Necrosciinae and Lonchodinae was not recovered in our analysis, which is supported by more mitochondrial data [32,33,39,45,52] than molecular data [40,46]. Heteropterygidae was sister to all remaining Oriophasmata based on the systematic analysis of transcriptome data [37,38]; however, this relationship was not recovered in the mitochondrial gene data or molecular marker data [5,41,45,51]. After the removal of Anisacanthini from the Heteropterygidae by Zompro [99], the phylogenetic relationships of the three subfamilies (Dataminae, Obriminae, and Heteropteryginae) have been controversial. Bank et al. [41] infer the phylogeny of Heteropterygidae covering the group’s overall diversity and supported Dataminae sister to (Heteropteryginae + Obriminae). However, molecular data do not provide a consistent conclusion [5,6,37,40,41,45,46]. Our analysis recovered Heteropterygidae as sister to the clade (Obriminae + Dataminae), which are consistent with many molecular studies [42,46]. Increasing the sample size and combining analysis of morphological features will allow further exploration of the relationships between the subfamilies. The Phasmatidae contain many subfamilies. Our study supported the monophyly of Phasmatidae, which is sister to Necrosciinae in the BI tree. This topology was recovered by Zhang et al. [100]. Phasmatidae contain many subfamilies, and Clitumninae has a rich diversity of species. Most molecular data studies have concluded that Clitumninae is a non-monophyletic group [5,6,40,56], and our study supports the paraphyly of this group.

## 5. Conclusions

Rearrangements are rare in the mitogenomes of Phasmatodea, and our discovery provides a new example for studying rearrangement mechanisms. The internal classification of Phasmatodea is constantly being revised. The newly sequenced samples in this study provide support for resolving the phylogenetic relationships of Lonchodidae, Phylliidae, and Heteropterygidae. With the further accumulation of molecular and morphological data, many internal classification contradictions will be resolved.

## Figures and Tables

**Table 1:** Best partitioning scheme and best-fitting models selected of mitochondrial 13 PCGs.

Nucleotide Sequence Alignments		
Subset	Subset Partitions	Best Model
Partition 1	ND6_codon1, ATP6_codon1, ND3_codon1, ND2_codon1, ATP8_codon1	GTR + I + G
Partition 2	Cytb_codon2, COX3_codon2, COX2_codon2, ND3_codon2, ND2_codon2, ATP6_codon2	GTR + I + G
Partition 3	ND2_codon3, ATP6_codon3, Cytb_codon3, ND6_codon3, ND3_codon3, ATP8_codon3	GTR + G
Partition 4	ATP8_codon2, ND2_codon2, ND6_codon2	GTR + I + G
Partition 5	COX1_codon1	GTR + I + G
Partition 6	COX1_codon2	TVM + I + G
Partition 7	COX1_codon3	GTR + I + G
Partition 8	Cytb_codon1, COX3_codon1, COX2_codon1	GTR + I + G
Partition 9	COX3_codon3, COX2_codon3	GTR + I + G
Partition 10	ND1_codon1, ND5_codon1	TRN + I + G
Partition 11	ND4L_codon2, ND5_codon2, ND1_codon2	GTR + G
Partition 12	ND4L_codon3, ND1_codon3, ND4_codon3, ND5_codon3	TVM + I + G
Partition 13	ND4_codon1, ND4L_codon1	TVM + I + G
Partition 14	ND4_codon2	GTR + I + G

## Data Availability

The data supporting the findings of this study are openly available from the National Center for Biotechnology Information at https://www.ncbi.nlm.nih.gov (accessed on 28 March 2023), accession numbers: OQ682523-OQ682531.

## Supplementary Materials

The following supporting information can be downloaded at: https://www.mdpi.com/article/10.3390/insects14050485/s1, Figure S1: The maps of seven newly sequenced mitogenomes. Mitochondrial genes located in the outer circle are encoded by the J-strand, and located in the inter circle are encoded by N-strand. The different kinds of tRNA are represented by amino acid abbreviations. GC content and GC skew are plotted as the deviation from the average value of the entire sequence; Figure S2: Nine histograms of the relative synonymous codon usage (RSCU) in newly sequenced mitogenomes. The X-axis shows synonymous codons, and the Y-axis shows RSCU values; Figure S3: The secondary structure of tRNAs in nine newly sequenced mitogenomes; Table S1: Information on the 58 species used for phylogenetic analysis [32,33,34,39,45,52,53,54,72,73]; Table S2: Codon saturation detection of 13 mtPCGs in DAMBE; Table S3: Location of features in newly sequenced mitogenomes; Table S4: Base composition of the newly sequenced mitogenomes; Table S5: Base composition of 13 PCGs in sequenced mitogenomes; Table S6: Codon usage of the 13 PCGs of the newly sequenced mitogenomes; Table S7: Amino acid use frequencies of proteins in the newly sequenced mitogenomes; Table S8: Start and stop codons of the 13 PCGs in the sequenced mitochondrial genes.
