# Supplementary material for: Nine Mitochondrial Genomes of Phasmatodea with Two Novel Mitochondrial Gene Rearrangements and Phylogeny
Source: Insects. 2023 May 22;14(5):485. doi: 10.3390/insects14050485 (PMC10231101; doi:10.3390/insects14050485)
Supplement: Supplementary file 1 [file insects-14-00485-s001.zip › insects-2359158-Supplementary.pdf]

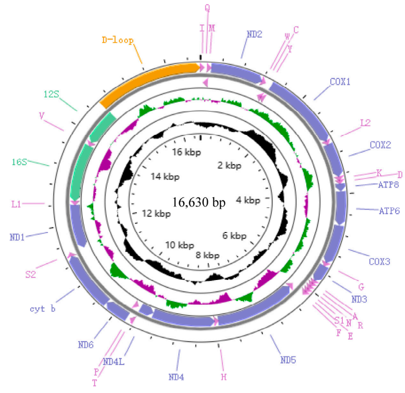

Marmessoidea bispinus OQ682527

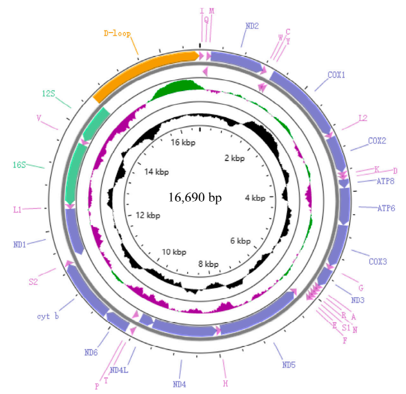

Phraortes lianzhouensis OQ682528

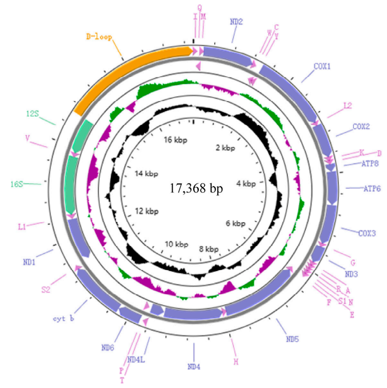

Sungaya inexpectata OQ682523

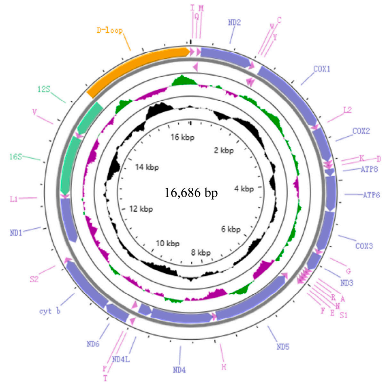

Pulchriphyllium bioculatum OQ682529

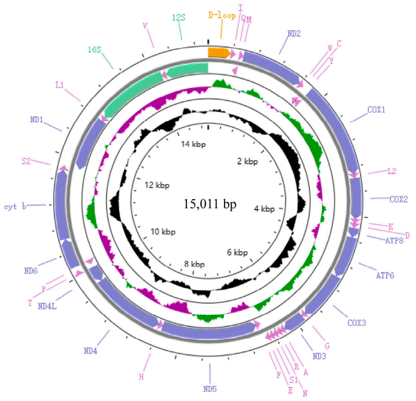

Lopaphus albopunctatus OQ682525

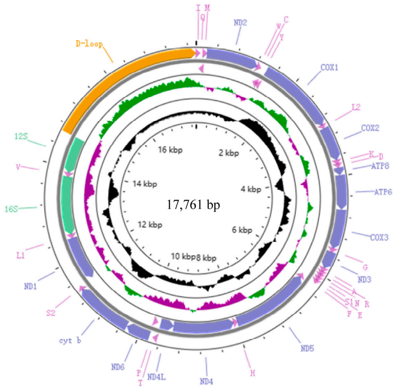

Lopaphus sphalerus OQ682526

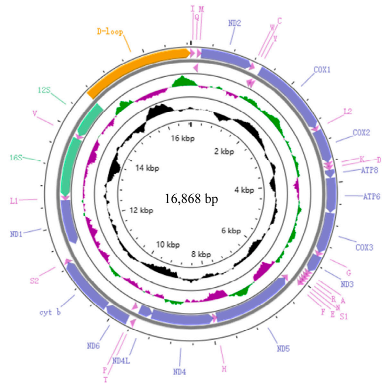

Pulchriphyllium giganteum OQ682530

CDS tRNA rRNA D-loop GC Skew+ GC Skew- GC Content

Figure S1. The maps of seven newly sequenced mitogenomes. Mitochondrial genes located in the outer circle are encoded by the J-strand, and located in the inter circle are encoded by N-strand. The different kinds of tRNA are represented by amino acid abbreviations. GC content and GC skew are plotted as the deviation from the average value of the entire sequence.

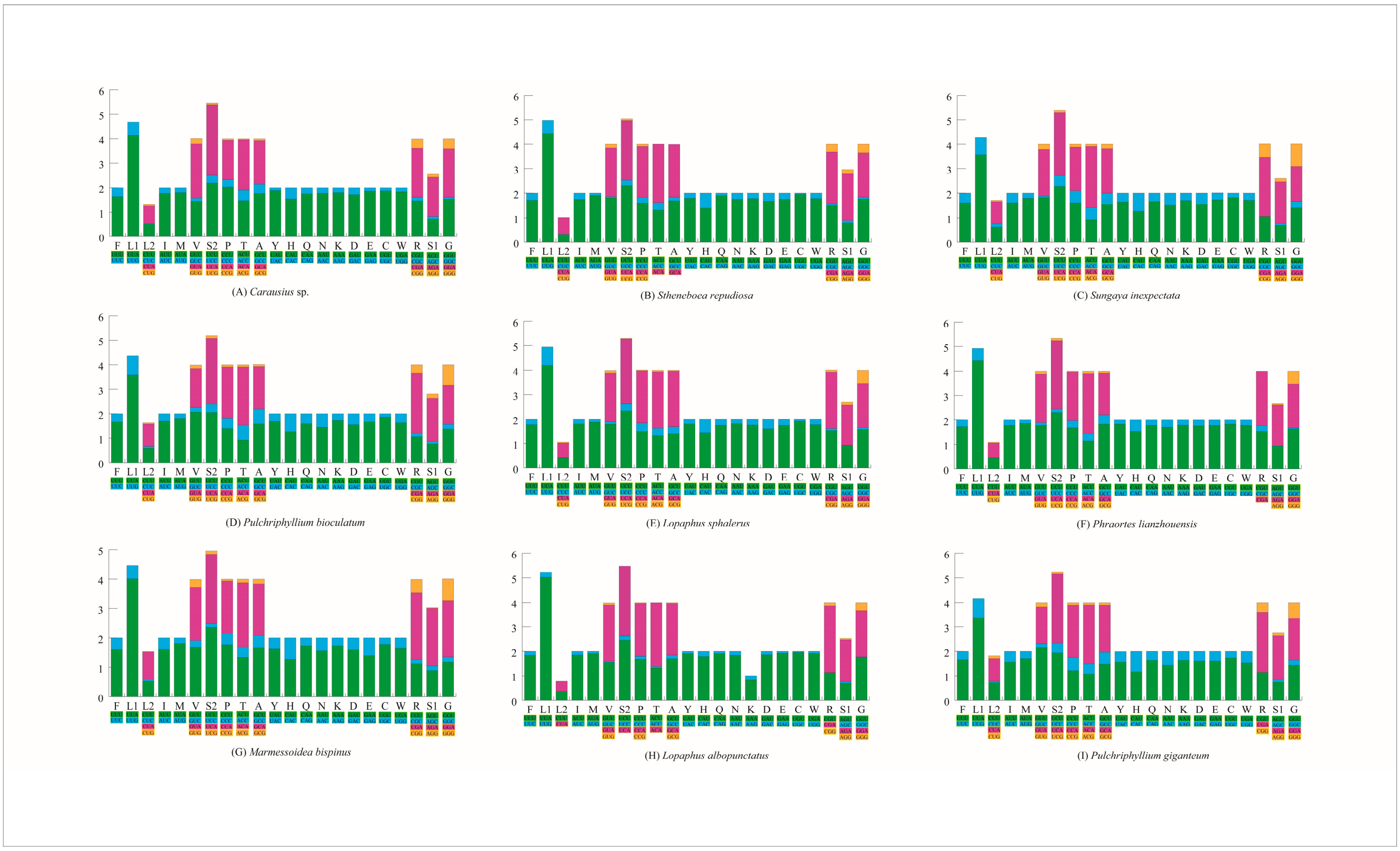

Figure S2. Nine histograms of the relative synonymous codon usage (RSCU) in newly sequenced mitogenomes. The X-axis shows synonymous codons, and the Y-axis shows RSCU values.

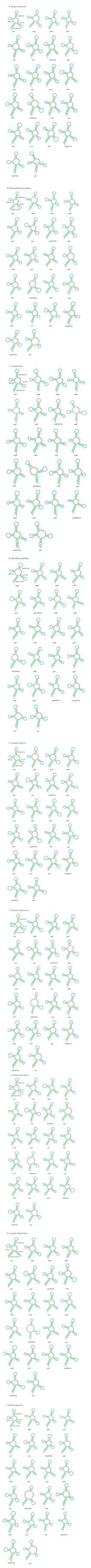

Figure S3. The secondary structure of tRNAs in nine newly sequenced mitogenomes.
