# Supplementary material for: Nine Mitochondrial Genomes of Phasmatodea with Two Novel Mitochondrial Gene Rearrangements and Phylogeny
Source: Insects. 2023 May 22;14(5):485. doi: 10.3390/insects14050485 (PMC10231101; doi:10.3390/insects14050485)
Supplement: Supplementary file 1 [file insects-14-00485-s001.zip › TableS1.pdf]

Table S1. Information on the 58 species used for phylogenetic analysis.

Family	Subfamily	Species	Length	Accession no.	Reference
Aschiphasmatidae	Aschiphasmatinae	*Nanhuaphasma hamicercum* Chen & He, 2002	14,921bp	MZ312646	[34]
		Orthomeria smaragdinum Redtenbacher, 1906	15,895 bp	MZ31264	[34]
Bacillidae	Bacillinae	*Bacillus rossius* Rossi, 1790	14,152bp	GU001956	[72]
		Bacillus atticus Brunner von Wattenwyl, 1882	14,141bp	GU001955	[72]
Heteropterygidae	Dataminae	*Orestes mouhotii* Bates, 1865	16,223 bp	AB477462	[39]
		Orestes guangxiensis Bi & Li, 1994	16,869 bp	MW450873	[45]
	Obriminae	*Aretaon asperrimus* Redtenbacher, 1906		SRR1172270	[52]
		Sungaya inexpectata Zompro, 1996	17,368 bp	OQ682523	This study
	Heteropteryginae	*Heteropteryx dilatat*e Parkinson, 1798	16,618 bp	AB477468	[39]
Lonchodidae	Lonchodinae	*Phraortes illepidus* Brunner von Wattenwyl, 1907	16,456 bp	AB477460	[39]
		Megalophasma granulatum Bi, 1995	15,275 bp	KY124331	[32]
		Phraortes sp. Miyako Island	14,913 bp	AB477465	[39]
		Phraortes sp. Iriomote Island	16,867 bp	AB477464	[39]
		Eurycantha calcarata Lucas, 1869	16,280 bp	MW915467	[54]
		Carasisus morosus Brunner von Wattenwyl, 1907		SRR3211828	[52]
		Carausius sp.	16,198 bp	OQ682524	This study
		Stheneboea repudiosa	16,376 bp	OQ682531	This study
		Brunner von Wattenwyl, 1907			
		Phraortes lianzhouensis Chen & He, 2008	16,690 bp	OQ682528	This study
	Necrosciinae	*Micadina phluctainoides* Rehn, 1904	16,507 bp	AB477466	[39]
		Sipyloidea sipylus Westwood, 1859	17,001 bp	AB477470	[39]
		Calvisia medogensis Bi, 1993	16,107 bp	KY124330	[32]
		Neohirasea japonica Haan, 1842	15,305 bp	AB477469	[39]
		Micadina brachyptera Liu & Cai, 1994	15,879 bp	MT025192	[33]
		Neohirasea stephanus Redtenbacher, 1908	18,021 bp	OL405132	Unpublished
		Lopaphus albopunctatus Chen & He, 2004	15,011 bp	OQ682525	This study
		Marmessoidea bispinus Burmeister, 1838	16,630 bp	OQ682527	This study
		Lopaphus sphalerus Redtenbacher, 1908	17,761 bp	OQ682526	This study
Phasmatidae	Megacraniinae	*Megacrania alpheus adan* Yamasaki, 1991	17,124 bp	AB477471	[39]
	Extatosomatinae	*Extatosoma tiaratum* Macleay, 1826	16,537 bp	AB642680	[53]
	Phasmatinae	*Acanthoxyla* sp.		SRR2089887	[52]
		Clitarchus hookeri White, 1846		SRR3080266	[52]
		Argosarchus horridus White, 1846		SRR2089902	[52]
	Clitumninae	*Entoria okinawaensis* Shiraki, 1935	16,910 bp	AB477459	[39]

		Phobaeticus serratipes Gray, 1835	16,182 bp	AB477467	[39]
		Ramulus hainanense Chen & He, 2002	15,590 bp	FJ156750	Unpublished
		Ramulus irregulariterdentatus Brunner von Wattenwyl, 1907	16,633 bp	AB477463	[39]
		Rumulus artemis Westwood, 1859		SRR1172422	[52]
		Medauroidea extradentata Brunner von Wattenwyl, 1907		SRR1172394	[52]
		Phryganistria guangxiensis Chen & He, 2008	17,005bp	MW450875	[45]
		Pharnaciini sp. NS-2020 Günther, 1953	15,192 bp	MT025193	[33]
	Pachymorphinae	*Niveaphasma anulata* Hutton, 1898		SRR2089878	[52]
		Tectarchus ovobsessus Salmon, 1954		SRR2089893	[52]
		Tectarchus salebrosus Hutton, 1899		SRR2089908	[52]
		Asteliaphasma jucundum Salmon, 1991		SRR2089913	[52]
		Spinotectarchus acornutus Hutton, 1899		SRR2089897	[52]
		Micrarchus hystriculeus Westwood, 1859		SRR1054191	[52]
		Micrarchus sp.		SRR1054193	[52]
Phylliidae	Phylliinae	*Cryptophyllium tibetense* Liu, 1993	18,248 bp	KX091862	Unpublished
		Cryptophyllium westwoodii Wood-Mason, 1875	17,222 bp	MW229063	Unpublished
		Pulchriphyllium bioculatum Gray, 1832	16,686 bp	OQ682529	This study
		Pulchriphyllium giganteum Hausleithner, 1984	16,868 bp	OQ682530	This study
		Pulchriphyllium giganteum Hausleithner, 1984	13,980 bp	AB477461	[39]
		Phyllium siccifolium Linnaeus, 1758		ERR392012	[52]
Pseudophasmatidae	Pseudophasmatinae	*Peruphasma schultei* Conle & Hennemann, 2005	15,896bp	MW450874	[45]
		Peruphasma schultei Conle & Hennemann, 2005		SRR1002984	[52]
Timematidae	Timematinae	*Timema californicum* Scudder, 1895	14,387 bp	DQ241799	[73]
Mantophasmatidae	Mantophasmatinae	*Sclerophasma paresisense* Klass, Picker, Damgaard, van Noort & Tojo, 2003	15,500 bp	DQ241798	[73]
Grylloblattidae		*Grylloblatta sculleni* Gurney, 1937	15,595 bp	DQ241796	[73]
