# Supplementary material for: Nine Mitochondrial Genomes of Phasmatodea with Two Novel Mitochondrial Gene Rearrangements and Phylogeny
Source: Insects. 2023 May 22;14(5):485. doi: 10.3390/insects14050485 (PMC10231101; doi:10.3390/insects14050485)
Supplement: Supplementary file 1 [file insects-14-00485-s001.zip › TableS2.pdf]

Table S2. Codon saturation detection of 13 mtPCGs in DAMBE.

(a) Results of first codon saturation detection.

Test of substitution saturation (Xia et al. 2003; Xia and Lemey 2009)

Analysis performed on all sites.

Testing whether the observed Iss is significantly lower than Iss.c.
IssSym is Iss.c assuming a symmetrical topology.
IssAsym is Iss.c assuming an asymmetrical topology.

NumOTU	Iss	Iss.cSym	T	DF	P	Iss.cAsym	T	DF	P
4	0.267	0.848	69.075	3206	0.0000	0.833	67.260	3206	0.0000
8	0.273	0.838	63.115	3206	0.0000	0.752	53.495	3206	0.0000
16	0.273	0.824	61.078	3206	0.0000	0.660	42.954	3206	0.0000
32	0.281	0.806	58.142	3206	0.0000	0.547	29.410	3206	0.0000

Note: two-tailed t-tests are used.

Interpretation of results:

	Significant Difference: Yes	Significant Difference: No
Iss < Iss.c	Little saturation	Substantial saturation
Iss > Iss.c	Useless sequences	Very poor for phylogenetics

(b) Results of second codon saturation detection.

Test of substitution saturation (Xia et al. 2003; Xia and Lemey 2009)

Analysis performed on all sites.

Testing whether the observed Iss is significantly lower than Iss.c.
IssSym is Iss.c assuming a symmetrical topology.
IssAsym is Iss.c assuming an asymmetrical topology.

NumOTU	Iss	Iss.cSym	T	DF	P	Iss.cAsym	T	DF	P
4	0.138	0.848	109.639	3206	0.0000	0.833	107.279	3206	0.0000
8	0.147	0.838	97.358	3206	0.0000	0.752	85.228	3206	0.0000
16	0.150	0.824	93.459	3206	0.0000	0.660	70.786	3206	0.0000
32	0.155	0.806	90.004	3206	0.0000	0.547	54.139	3206	0.0000

Note: two-tailed t-tests are used.

Interpretation of results:

	Significant Difference: Yes	Significant Difference: No
Iss < Iss.c	Little saturation	Substantial saturation
Iss > Iss.c	Useless sequences	Very poor for phylogenetics

(c) Results of third codon saturation detection.

Test of substitution saturation (Xia et al. 2003; Xia and Lemey 2009)

Analysis performed on all sites.

Testing whether the observed Iss is significantly lower than Iss.c.
IssSym is Iss.c assuming a symmetrical topology.
IssAsym is Iss.c assuming an asymmetrical topology.

NumOTU	Iss	Iss.cSym	T	DF	P	Iss.cAsym	T	DF	P
4	0.598	0.848	27.294	3206	0.0000	0.833	25.627	3206	0.0000
8	0.621	0.838	24.595	3206	0.0000	0.752	14.822	3206	0.0000
16	0.619	0.824	24.967	3206	0.0000	0.660	5.026	3206	0.0000
32	0.625	0.806	23.528	3206	0.0000	0.547	10.111	3206	0.0000

Note: two-tailed t-tests are used.

Interpretation of results:

	Significant Difference: Yes	Significant Difference: No
Iss < Iss.c	Little saturation	Substantial saturation
Iss > Iss.c	Useless sequences	Very poor for phylogenetics
