# Supplementary material for: Nine Mitochondrial Genomes of Phasmatodea with Two Novel Mitochondrial Gene Rearrangements and Phylogeny
Source: Insects. 2023 May 22;14(5):485. doi: 10.3390/insects14050485 (PMC10231101; doi:10.3390/insects14050485)
Supplement: Supplementary file 1 [file insects-14-00485-s001.zip › TableS3.pdf]

Table S3. Location of features in newly sequenced mitogenomes.

A. *Sungaya inexpectata*

Gene	Strand	Position	Length (nuc.)	Anticodon	Start codon	Stop codon	Intergenic nucleotides
$tRNA^{Ile}$	+	1-69	69	ATC			-3
$tRNA^{Gln}$	-	67-135	69	CAA			-1
$tRNA^{Met}$	+	135-200	66	ATG			0
ND2	+	201-1214	1014		ATT	TAA	-2
$tRNA^{Trp}$	+	1213-1279	67	TGA			-8
$tRNA^{Cys}$	-	1272-1336	65	TGC			+3
$tRNA^{Tyr}$	-	1340-1403	64	TAC			+1
COX1	+	1405-2938	1534		ATG	T	0
$tRNA^{Leu2(UUA)}$	+	2939-3002	64	TTA			0
COX2	+	3003-3672	670		ATA	T	0
$tRNA^{Lys}$	+	3673-3742	70	AAG			-1
$tRNA^{Asp}$	+	3742-3806	65	GAC			1
ATP8	+	3808-3963	156		ATT	TAA	-4
ATP6	+	3960-4634	675		ATA	TAA	-1
COX3	+	4634-5419	786		ATG	TAA	-1
$tRNA^{Gly}$	+	5419-5482	64	GGA			0
ND3	+	5483-5836	354		ATT	TAG	-2
$tRNA^{Ala}$	+	5835-5899	65	GCA			0
$tRNA^{Arg}$	+	5900-5968	69	CGA			+4
$tRNA^{Asn}$	+	5973-6036	64	AAC			0
$tRNA^{Ser1}$	+	6037-6104	68	AGC			+1
$tRNA^{Glu}$	+	6106-6169	64	GAA			-2
$tRNA^{Phe}$	-	6168-6233	66	TTC			-1
ND5	-	6233-7956	1724		ATT	T	0
$tRNA^{His}$	-	7957-8021	65	CAC			-1
ND4	-	8021-9352	1332		ATG	TAA	-7
ND4L	-	9346-9630	285		ATT	TAA	+8
$tRNA^{Thr}$	+	9639-9703	65	ACA			0
$tRNA^{Pro}$	-	9704-9771	68	CCA			+1
ND6	+	9773-10255	483		ATT	TAA	-1
Cytb	+	10255-11388	1134		ATG	TAG	-2
$tRNA^{Ser2}$	+	11387-11454	68	TCA			-2
ND1	-	11453-12421	969		TTG	TAA	0
$tRNA^{Leu1(CUA)}$	-	12422-12487	66	CTA			0
16S rRNA	-	12488-13792	1305				0
$tRNA^{Val}$	-	13793-13862	70	GTA			0
12S rRNA	-	13863-14640	778				0
CR		14641-17368	2728				

B. *Pulchriphyllium bioculatum*

Gene	Strand	Position	Length (nuc.)	Anticodon	Start codon	Stop codon	Intergenic nucleotides
tRNA^{Ile}	+	1-66	66	ATC			-3
tRNA^{Gln}	-	64-131	68	CAA			-1
tRNA^{Met}	+	131-195	65	ATG			0
ND2	+	196-1189	994		ATT	T	-2
tRNA^{Trp}	+	1190-1252	63	TGA			-8
tRNA^{Cys}	-	1245-1306	62	TGC			+1
tRNA^{Tyr}	-	1308-1374	67	TAC			+1
COX1	+	1376-2909	1534		ATG	T	0
tRNA$^{Leu2(UUA)}$	+	2910-2973	64	TTA			0
COX2	+	2974-3625	652		ATT	T	0
tRNA^{Lys}	+	3626-3694	69	AAG			-1
tRNA^{Asp}	+	3694-3759	66	GAC			0
ATP8	+	3760-3912	153		ATA	TAA	-4
ATP6	+	3909-4583	675		ATA	TAA	-1
COX3	+	4583-5365	783		ATG	TAA	-1
tRNA^{Gly}	+	5365-5426	62	GGA			0
ND3	+	5427-5778	352		ATA	T	0
tRNA^{Ala}	+	5779-5841	63	GCA			0
tRNA^{Arg}	+	5842-5907	66	CGA			+2
tRNA^{Asn}	+	5910-5973	64	AAC			-2
tRNA^{Ser1}	+	5972-6033	62	AGC			+1
tRNA^{Glu}	+	6033-6098	66	GAA			-2
tRNA^{Phe}	-	6097-6163	67	TTC			0
ND5	-	6164-7885	1722		ATA	TAG	0
tRNA^{His}	-	7886-7950	65	CAC			-3
ND4	-	7948-9277	1330		ATG	T	-7
ND4L	-	9271-9555	285		ATT	TAA	+8
tRNA^{Thr}	+	9564-9624	61	ACA			0
tRNA^{Pro}	-	9625-9689	65	CCA			+1
ND6	+	9691-10173	483		ATT	TAA	-1
Cytb	+	10173-11297	1125		ATG	TAA	0
tRNA^{Ser2}	+	11298-11357	60	TCA			+198
ND1	-	11556-12521	966		ATA	TAA	+3
tRNA$^{Leu1(CUA)}$	-	12525-12590	66	CTA			0
16S rRNA	-	12591-13859	1269				0
tRNA^{Val}	-	13860-13927	68	GTA			0
12S rRNA	-	13928-14710	783				0
CR		14711-16868	2158				

C. *Carausius* sp.

Gene	Strand	Position	Length (nuc.)	Anticodon	Start codon	Stop codon	Intergenic nucleotides
$tRNA^{Ile}$	+	1-66	66	ATC			-3
$tRNA^{Gln}$	-	64-133	70	CAA			-1
$tRNA^{Met}$	+	133-198	66	ATG			0
ND2	+	199-1185	987		ATC	TAA	-2
$tRNA^{Trp}$	+	1184-1249	66	TGA			-8
$tRNA^{Cys}$	-	1242-1302	61	TGC			0
$tRNA^{Tyr}$	-	1303-1366	64	TAC			+1
COX1	+	1368-2901	1534		ATG	T	0
$tRNA^{Leu2(UUA)}$	+	2902-2967	66	TTA			0
COX2	+	2968-3637	670		ATT	T	0
$tRNA^{Lys}$	+	3638-3707	70	AAG			-1
$tRNA^{Asp}$	+	3707-3774	68	GAC			0
ATP8	+	3775-3930	156		ATA	TAA	-4
ATP6	+	3927-4592	666		ATA	TAA	-1
COX3	+	4592-5374	783		ATG	TAA	-1
$tRNA^{Gly}$	+	5374-5435	62	GGA			0
ND3	+	5436-5786	351		ATT	TAA	-2
$tRNA^{Arg}$	+	5785-5848	64	CGA			+2
$tRNA^{Ala}$	+	5851-5913	63	GCA			+19
$tRNA^{Asn}$	+	5933-5997	65	AAC			-1
$tRNA^{Ser1}$	+	5997-6059	63	AGC			-1
$tRNA^{Glu}$	+	6059-6124	66	GAA			-2
$tRNA^{Phe}$	-	6123-6184	62	TTC			0
ND5	-	6185-7905	1721		ATT	TA	0
$tRNA^{His}$	-	7906-7967	62	CAC			+1
ND4	-	7969-9300	1332		ATG	TAG	-7
ND4L	-	9294-9578	285		ATA	TAA	+8
$tRNA^{Thr}$	+	9587-9650	64	ACA			0
$tRNA^{Pro}$	-	9651-9714	64	CCA			+1
ND6	+	9716-10186	471		ATT	TAA	-1
Cytb	+	10186-11319	1134		ATG	TAA	-2
$tRNA^{Ser2}$	+	11318-11383	66	TCA			-2
ND1	-	11382-12347	966		TTG	TAA	0
$tRNA^{Leu1(CUA)}$	-	12348-12413	66	CTA			0
16S rRNA	-	12414-13676	1263				0
$tRNA^{Val}$	-	13677-13747	71	GTA			0
12S rRNA	-	13748-14537	790				0
CR		14538-16198	1661				

D. *Stheneboea repudiosa*

Gene	Strand	Position	Length (nuc.)	Anticodon	Start codon	Stop codon	Intergenic nucleotides
$tRNA^{Gln}$	-	1-69	69	CAA			-1
$tRNA^{Met}$	+	69-134	66	ATG			0
ND2	+	135-1122	988		ATA	T	0
$tRNA^{Trp}$	+	1123-1187	65	TGA			-8
$tRNA^{Cys}$	-	1180-1241	62	TGC			0
$tRNA^{Tyr}$	-	1242-1309	68	TAC			+1
COX1	+	1311-2844	1534		ATG	T	0
$tRNA^{Leu2(UUA)}$	+	2845-2908	64	TTA			0
COX2	+	2909-3577	669		ATA	TAA	0
$tRNA^{Lys}$	+	3578-3648	71	AAG			0
$tRNA^{Asp}$	+	3649-3712	64	GAC			+1
ATP8	+	3714-3869	156		ATA	TAA	-4
ATP6	+	3866-4537	672		ATA	TAA	-1
COX3	+	4537-5325	789		ATG	TAA	-1
$tRNA^{Gly}$	+	5325-5387	63	GGA			0
ND3	+	5388-5739	352		ATT	T	0
$tRNA^{Ala}$	+	5740-5804	65	GCA			-1
$tRNA^{Arg}$	+	5804-5868	65	CGA			+2
$tRNA^{Asn}$	+	5871-5935	65	AAC			0
$tRNA^{Ser1}$	+	5936-5997	62	AGC			0
$tRNA^{Glu}$	+	5998-6064	67	GAA			-3
$tRNA^{Phe}$	-	6062-6125	64	TTC			0
ND5	-	6126-7848	1723		ATT	T	0
$tRNA^{His}$	-	7849-7912	64	CAC			0
ND4	-	7913-9239	1327		ATG	T	-7
ND4L	-	9233-9520	288		ATG	TAA	+5
$tRNA^{Thr}$	+	9526-9588	63	ACA			0
$tRNA^{Pro}$	-	9589-9657	69	CCA			+1
ND6	+	9659-10135	477		ATA	TAA	-1
Cytb	+	10135-11268	1134		ATG	TAG	-2
$tRNA^{Ser2}$	+	11267-11331	65	TCA			+7
ND1	-	11339-12298	960		TTG	TAA	0
$tRNA^{Leu1(CUA)}$	-	12299-12365	67	CTA			0
16S rRNA	-	12366-13629	1264				0
$tRNA^{Val}$	-	13630-13697	68	GTA			0
12S rRNA	-	13698-14469	772				0
CR		14470-15449	980				0
$tRNA^{Ile}$	-	15450-15514	65	ATC			0
CR		15515-16376	862				

E. *Lopaphus sphalerus*

Gene	Strand	Position	Length (nuc.)	Anticodon	Start codon	Stop codon	Intergenic nucleotides
$tRNA^{Ile}$	+	1-67	67	ATC			+2
$tRNA^{Gln}$	-	70-138	69	CAA			-1
$tRNA^{Met}$	+	138-205	68	ATG			0
ND2	+	206-1225	1020		ATT	TAA	-2
$tRNA^{Trp}$	+	1224-1290	67	TGA			-8
$tRNA^{Cys}$	-	1283-1346	64	TGC			+2
$tRNA^{Tyr}$	-	1349-1417	69	TAC			+1
COX1	+	1419-2952	1534		ATG	T	0
$tRNA^{Leu2(UUA)}$	+	2953-3018	66	TTA			0
COX2	+	3019-3688	670		ATT	T	0
$tRNA^{Lys}$	+	3689-3758	70	AAG			-1
$tRNA^{Asp}$	+	3758-3828	71	GAC			0
ATP8	+	3829-3987	159		ATA	TAA	-4
ATP6	+	3984-4658	675		ATA	TAA	-1
COX3	+	4658-5444	787		ATG	T	0
$tRNA^{Gly}$	+	5445-5509	65	GGA			0
ND3	+	5510-5861	352		ATA	T	0
$tRNA^{Ala}$	+	5862-5930	69	GCA			0
$tRNA^{Arg}$	+	5931-5995	65	CGA			+2
$tRNA^{Asn}$	+	5993-6058	66	AAC			0
$tRNA^{Ser1}$	+	6059-6126	68	AGC			+1
$tRNA^{Glu}$	+	6128-6192	65	GAA			-2
$tRNA^{Phe}$	-	6191-6257	67	TTC			0
ND5	-	6258-7980	1723		ATT	T	0
$tRNA^{His}$	-	7981-8045	65	CAC			-3
ND4	-	8046-9377	1332		ATG	TAA	-7
ND4L	-	9371-9661	285		ATT	TAA	+2
$tRNA^{Thr}$	+	9664-9730	67	ACA			+5
$tRNA^{Pro}$	-	9736-9805	70	CCA			+1
ND6	+	9807-10286	480		ATA	TAA	-1
Cytb	+	10286-11419	1134		ATG	TAA	-1
$tRNA^{Ser2}$	+	11419-11483	65	TCA			-2
ND1	-	11482-12447	966		ATA	TAA	+3
$tRNA^{Leu1(CUA)}$	-	12451-12521	71	CTA			0
16S rRNA	-	12522-13804	1283				0
$tRNA^{Val}$	-	13805-13874	70	GTA			0
12S rRNA	-	13875-14654	780				0
CR		14655-17761	3107				

F. *Phraortes lianzhouensis*

Gene	Strand	Position	Length (nuc.)	Anticodon	Start codon	Stop codon	Intergenic nucleotides
tRNA^Ile^	+	1-66	66	ATC			+2
tRNA^Gln^	-	69-137	69	CAA			-1
tRNA^Met^	+	137-200	64	ATG			0
ND2	+	201-1193	993		ATC	TAA	-2
tRNA^Trp^	+	1192-1256	65	TGA			-8
tRNA^Cys^	-	1249-1310	62	TGC			-1
tRNA^Tyr^	-	1310-1373	64	TAC			+1
COX1	+	1375-2908	1534		ATG	T	0
tRNA^Leu2(UUA)^	+	2909-2973	65	TTA			0
COX2	+	2974-3640	667		ATA	T	0
tRNA^Lys^	+	3641-3710	70	AAG			-1
tRNA^Asp^	+	3710-3774	65	GAC			+1
ATP8	+	3776-3934	159		ATG	TAA	-4
ATP6	+	3931-4605	675		ATA	TAA	-1
COX3	+	4605-5391	787		ATG	T	0
tRNA^Gly^	+	5392-5456	65	GGA			0
ND3	+	5457-5810	354		ATT	TAG	-2
tRNA^Ala^	+	5809-5873	65	GCA			-1
tRNA^Arg^	+	5873-5940	68	CGA			+3
tRNA^Asn^	+	5944-6008	65	AAC			0
tRNA^Ser1^	+	6009-6075	67	AGC			0
tRNA^Glu^	+	6076-6139	64	GAA			-2
tRNA^Phe^	-	6138-6201	64	TTC			0
ND5	-	6202-7925	1724		ATT	T	0
tRNA^His^	-	7926-7993	68	CAC			-1
ND4	-	7993-9318	1326		ATA	TAG	-7
ND4L	-	9312-9596	285		ATA	TAA	+8
tRNA^Thr^	+	9605-9669	65	ACA			0
tRNA^Pro^	-	9670-9734	65	CCA			+1
ND6	+	9736-10206	471		ATA	TAA	-1
Cytb	+	10206-11336	1131		ATG	TAG	-2
tRNA^Ser2^	+	11335-11402	68	TCA			+1
ND1	-	11404-12367	964		ATT	T	+3
tRNA^Leu1(CUA)^	-	12371-12438	68	CTA			0
16S rRNA	-	12439-13719	1281				0
tRNA^Val^	-	13720-13788	69	GTA			0
12S rRNA	-	13789-14568	780				0
CR		14569-16690	2122				

G. *Marmessoidea bispinus*

Gene	Strand	Position	Length (nuc.)	Anticodon	Start codon	Stop codon	Intergenic nucleotides
tRNA^{Ile}	+	1-67	67	ATC			0
tRNA^{Gln}	-	68-133	66	CAA			-1
tRNA^{Met}	+	133-201	69	ATG			0
ND2	+	202-1200	999		ATT	TAA	-2
tRNA^{Trp}	+	1199-1264	66	TGA			-8
tRNA^{Cys}	-	1257-1318	62	TGC			+3
tRNA^{Tyr}	-	1322-1388	67	TAC			+1
COX1	+	1390-2923	1534		ATG	T	0
tRNA$^{Leu2(UUA)}$	+	2924-2988	65	TTA			0
COX2	+	2989-3658	670		ATA	T	0
tRNA^{Lys}	+	3659-3729	71	AAG			-1
tRNA^{Asp}	+	3729-3794	66	GAC			+1
ATP8	+	3796-3954	159		ATG	TAA	-4
ATP6	+	3951-4619	669		ATA	TAA	-1
COX3	+	4619-5407	789		ATG	TAA	-1
tRNA^{Gly}	+	5407-5470	64	GGA			0
ND3	+	5471-5822	352		ATT	T	0
tRNA^{Ala}	+	5823-5886	64	GCA			0
tRNA^{Arg}	+	5887-5950	64	CGA			+4
tRNA^{Asn}	+	5955-6020	66	AAC			0
tRNA^{Ser1}	+	6021-6088	68	AGC			0
tRNA^{Glu}	+	6089-6153	65	GAA			+2
tRNA^{Phe}	-	6156-6221	66	TTC			+9
ND5	-	6231-7943	1713		ATT	TAA	0
tRNA^{His}	-	7944-8007	64	CAC			+1
ND4	-	8009-9340	1332		GTG	TAG	-7
ND4L	-	9334-9624	291		ATG	TAA	+7
tRNA^{Thr}	+	9632-9695	64	ACA			0
tRNA^{Pro}	-	9696-9760	65	CCA			+2
ND6	+	9763-10245	483		ATT	TAA	-1
Cytb	+	10245-11381	1137		ATG	TAA	+1
tRNA^{Ser2}	+	11383-11449	67	TCA			-2
ND1	-	11448-12413	966		ATA	TAA	+3
tRNA$^{Leu1(CUA)}$	-	12417-12482	66	CTA			0
16S rRNA	-	12483-13762	1280				0
tRNA^{Val}	-	13763-13831	69	GTA			0
12S rRNA	-	13832-14598	767				0
CR		14599-16630	2032				0

H. *Lopaphus albopunctatus*

Gene	Strand	Position	Length (nuc.)	Anticodon	Start codon	Stop codon	Intergenic nucleotides
$tRNA^{Ile}$	+	1-68	68	ATC			0
$tRNA^{Gln}$	-	69-137	69	CAA			0
$tRNA^{Met}$	+	138-204	67	ATG			0
ND2	+	205-1218	1014		ATT	TAA	-2
$tRNA^{Trp}$	+	1217-1282	66	TGA			-8
$tRNA^{Cys}$	-	1275-1337	63	TGC			+4
$tRNA^{Tyr}$	-	1342-1411	70	TAC			+1
COX1	+	1413-2946	1534		ATG	T	0
$tRNA^{Leu2(UUA)}$	+	2947-3011	65	TTA			0
COX2	+	3012-3681	670		ATA	T	0
$tRNA^{Lys}$	+	3682-3752	71	AAG			-1
$tRNA^{Asp}$	+	3752-3819	68	GAC			0
ATP8	+	3820-3978	159		ATA	TAA	-4
ATP6	+	3975-4649	675		ATA	TAA	-1
COX3	+	4649-5437	789		ATG	TAA	-1
$tRNA^{Gly}$	+	5437-5501	65	GGA			0
ND3	+	5502-5853	352		ATT	T	0
$tRNA^{Ala}$	+	5854-5918	65	GCA			0
$tRNA^{Arg}$	+	5919-5986	68	CGA			0
$tRNA^{Asn}$	+	5987-6052	66	AAC			0
$tRNA^{Ser1}$	+	6053-6120	68	AGC			0
$tRNA^{Glu}$	+	6121-6185	65	GAA			-2
$tRNA^{Phe}$	-	6184-6249	66	TTC			-1
ND5	-	6249-7973	1725		ATG	TAA	0
$tRNA^{His}$	-	7974-8037	64	CAC			-1
ND4	-	8037-9368	1332		ATG	TAA	-7
ND4L	-	9362-9646	285		ATT	TAA	+8
$tRNA^{Thr}$	+	9655-9718	64	ACA			+6
$tRNA^{Pro}$	-	9725-9790	66	CCA			+1
ND6	+	9792-10265	474		ATA	TAA	-1
Cytb	+	10265-11398	1134		ATG	TAG	-2
$tRNA^{Ser2}$	+	11397-11464	68	TCA			-2
ND1	-	11463-12428	966		ATA	TAA	+3
$tRNA^{Leu1(CUA)}$	-	12432-12502	71	CTA			0
16S rRNA	-	12504-13789	1286				0
$tRNA^{Val}$	-	13790-13859	70	GTA			0
12S rRNA	-	13859-14625	767				0
CR		14626-15011	386				

I. *Pulchriphyllium giganteum*

Gene	Strand	Position	Length (nuc.)	Anticodon	Start codon	Stop codon	Intergenic nucleotides
tRNA^{Ile}	+	1-65	65	ATC			-3
tRNA^{Gln}	-	63-131	69	CAA			-1
tRNA^{Met}	+	131-194	64	ATG			0
ND2	+	195-1193	999		ATA	TAA	-2
tRNA^{Trp}	+	1192-1256	65	TGA			-8
tRNA^{Cys}	-	1249-1310	62	TGC			+1
tRNA^{Tyr}	-	1312-1375	64	TAC			+1
COX1	+	1377-2910	1534		ATG	T	0
tRNA$^{Leu2(UUA)}$	+	2911-2973	63	TTA			0
COX2	+	2974-3640	667		ATA	T	0
tRNA^{Lys}	+	3641-3709	69	AAG			-1
tRNA^{Asp}	+	3709-3772	64	GAC			0
ATP8	+	3773-3925	153		ATA	TAA	-4
ATP6	+	3922-4593	672		ATA	TAA	-1
COX3	+	4593-5375	783		ATG	TAA	-1
tRNA^{Gly}	+	5375-5436	62	GGA			0
ND3	+	5437-5788	352		ATA	T	0
tRNA^{Ala}	+	5789-5854	66	GCA			0
tRNA^{Arg}	+	5855-5918	64	CGA			+2
tRNA^{Asn}	+	5921-5985	65	AAC			0
tRNA^{Ser1}	+	5986-6048	63	AGC			0
tRNA^{Glu}	+	6049-6113	65	GAA			-2
tRNA^{Phe}	-	6112-6176	65	TTC			-1
ND5	-	6176-7894	1719		ATT	TAA	0
tRNA^{His}	-	7895-7957	63	CAC			0
ND4	-	7958-9287	1330		ATG	T	-7
ND4L	-	9281-9565	285		ATG	TAA	-2
tRNA^{Thr}	+	9574-9636	63	ACA			0
tRNA^{Pro}	-	9637-9698	62	CCA			+1
ND6	+	9700-10185	486		ATA	TAA	-1
Cytb	+	10185-11309	1125		ATG	TAA	+2
tRNA^{Ser2}	+	11312-11373	62	TCA			+15
ND1	-	11389-12354	966		ATA	TAG	+3
tRNA$^{Leu1(CUA)}$	-	12358-12423	66	CTA			0
16S rRNA	-	12424-13517	1292				-2
tRNA^{Val}	-	13716-13784	69	GTA			0
12S rRNA	-	13785-14544	760				0
CR		14545-16686	2142				
