# Supplementary material for: Nine Mitochondrial Genomes of Phasmatodea with Two Novel Mitochondrial Gene Rearrangements and Phylogeny
Source: Insects. 2023 May 22;14(5):485. doi: 10.3390/insects14050485 (PMC10231101; doi:10.3390/insects14050485)
Supplement: Supplementary file 1 [file insects-14-00485-s001.zip › TableS4.pdf]

Table S4. Base composition of the newly sequenced mitogenomes.

Family	Genera	Species	Region	Whole genome	PCGs		tRNA		rRNA	Control Region
			Strand	+	+	-	+	-	-	+
Heteropterygidae	*Obrimini*	*Sungaya inexpectata*	Length(bp)	17,368	6,804	4,308	928	533	2083	2728
			A+T%	74.3	72	76.3	77.8	73.3	76.6	74.6
			AT-Skew	0.235	0.123	-0.418	0.118	-0.151	-0.282	0.231
			GC-Skew	-0.203	-0.194	0.269	-0.01	0.324	0.309	-0.083
Phylliidae	*Pulchriphyllium*	*Pulchriphyllium bioculatum*	Length(bp)	16,868	6,750	4,302	897	528	2052	2158
			A+T%	74.9	73.3	76.8	80.9	75	77.8	71.4
			AT-Skew	0.223	0.147	-0.436	0.125	-0.157	-0.322	-0.008
			GC-Skew	-0.255	-0.228	0.305	0.041	0.424	0.335	-0.220
Phylliidae	*Pulchriphyllium*	*Pulchriphyllium giganteum*	Length(bp)	16686	6768	4299	897	528	2052	2142
			A+T%	73.8	72.2	75.9	79	74.9	77.3	68.6
			AT-Skew	0.236	0.147	-0.449	0.142	-0.167	-0.289	0.064
			GC-Skew	-0.22	-0.19	0.265	0.016	0.142	0.270	-0.223
Lonchodidae	*Carausius*	*Carausius* sp.	Length(bp)	16,198	6,750	4,302	915	520	2053	1661
			A+T%	77.4	75.7	77.7	80	78	79.2	80.3
			AT-Skew	0.160	0.058	-0.315	0.082	-0.133	-0.202	0.163
			GC-Skew	-0.211	-0.176	0.256	-0.016	0.368	0.28	-0.214
Lonchodidae	*Lopaphus*	*Lopaphus sphalerus*	Length(bp)	17,761	6,807	4,305	940	545	2063	3107
			A+T%	78.2	75.8	79.1	81.1	79.5	79.5	80.8
			AT-Skew	0.217	0.106	-0.396	0.085	-0.104	-0.265	0.230
			GC-Skew	-0.152	-0.13	0.253	0.067	0.411	0.294	0.021
Lonchodidae	*Phraortes*	*Phraortes lianzhouensis*	Length(bp)	16,690	6,768	4,296	922	529	2061	2122

			A+T%	77.7	75.5	78.5	79.5	78	78.4	81.7
			AT-Skew	0.168	0.075	-0.366	0.074	-0.141	-0.225	0.053
			GC-Skew	-0.121	-0.103	0.216	0.037	0.414	0.251	0.191
Lonchodidae	*Marmessoidea*	*Marmessoidea bispinus*	Length(bp)	16,630	6,789	4,302	926	525	2047	2032
			A+T%	75.8	73	77.3	79.8	79.6	78.6	76.3
			AT-Skew	0.189	0.101	-0.353	0.08	-0.105	-0.229	0.151
			GC-Skew	-0.188	-0.154	0.235	0.037	0.421	0.277	-0.178
Lonchodidae	*Lopaphus*	*Lopaphus albopunctatus*	Length(bp)	15,011	6,798	4,308	934	539	2053	386
			A+T%	79.3	77.8	81.0	80.3	80.5	80.7	76.2
			AT-Skew	0.176	0.072	-0.33	0.053	-0.078	-0.217	0.412
			GC-Skew	-0.118	-0.066	0.169	0.054	0.39	0.259	0.042
Lonchodidae	*Stheneboea*	*Stheneboea repudiosa*	Length(bp)	16,376	6,774	4,296	845	596	2036	1842
			A+T%	78.5	76.2	79.1	82.1	78.4	79.8	82.1
			AT-Skew	0.196	0.102	-0376	0.115	-0.122	-0.249	0.113
			GC-Skew	-0.237	-0.186	0.295	-0.007	0.442	0.301	-0.263
