# Supplementary material for: Nine Mitochondrial Genomes of Phasmatodea with Two Novel Mitochondrial Gene Rearrangements and Phylogeny
Source: Insects. 2023 May 22;14(5):485. doi: 10.3390/insects14050485 (PMC10231101; doi:10.3390/insects14050485)
Supplement: Supplementary file 1 [file insects-14-00485-s001.zip › TableS5.pdf]

Table S5. Base composition of 13 PCGs in sequenced mitogenomes.

Species	Gene	ATP6	ATP8	COX1	COX2	COX3	Cytb	ND1	ND2	ND3	ND4	ND4L	ND5	ND6
	Strand	+	+	+	+	+	+	-	+	+	-	-	-	+
Sungaya inexpectata	length	675	156	1534	670	786	1134	969	1014	354	1311	285	1724	483
	A+T%	74.6	83.3	67.5	70.7	69.5	69	76.6	78.5	71.8	75.6	78.3	76	78.7
	AT-Skew	0.123	0.277	0.035	0.143	0.077	0.098	-0.456	0.214	0.142	-0.407	-0.48	-0.391	0.189
	GC-Skew	-0.24	-0.385	-0.09	-0.184	-0.1	-0.179	0.321	-0.321	-0.32	0.21	0.258	0.278	-0.476
Pulchriphyllium bioculatum	length	675	153	1534	652	783	1125	966	996	352	1330	285	1722	483
	A+T%	75.7	84.9	68.4	72.4	71.1	71.7	75.9	78.2	75.9	76.4	81.4	76.9	78.3
	AT-Skew	0.166	0.246	0.065	0.191	0.131	0.152	-0.457	0.168	0.101	-0.459	-0.431	-0.407	0.254
	GC-Skew	-0.293	-0.739	-0.117	-0.189	-0.124	-0.283	0.33	-0.309	-0.365	0.255	0.283	0.333	-0.371
Pulchriphyllium giganteum	length	672	153	1534	667	783	1125	966	999	352	1330	285	1719	486
	A+T%	74.8	83	67.9	71.8	70.6	69.9	75.5	76.6	71.8	75.1	79	76.4	78.2
	AT-Skew	0.141	0.307	0.068	0.215	0.125	0.13	-0.496	0.18	0.123	-0.459	-0.467	-0.412	0.247
	GC-Skew	-0.243	-0.077	-0.073	-0.138	-0.078	-0.257	0.297	-0.305	-0.273	0.19	0.433	0.283	-0.472
Carausius sp.	length	666	156	1534	670	783	1134	966	987	351	1332	285	1721	471
	A+T%	77.4	82.7	70.3	74.5	71.2	74.9	77.8	82.2	78.4	77.2	80.1	77.6	84.1
	AT-Skew	0.062	0.163	-0.006	0.106	0.045	-0.02	-0.358	0.132	0.069	-0.331	-0.339	-0.268	0.152
	GC-Skew	-0.267	-0.407	-0.081	-0.17	-0.186	-0.123	0.278	-0.216	-0.368	0.287	0.276	0.214	-0.413
Stheneboea repudiosa	length	672	156	1534	669	789	1134	960	990	354	1327	288	1723	477
	A+T%	79.3	87.2	71.5	75.4	73	74.1	78.8	81.9	78	79.2	81.8	78.7	82.6
	AT-Skew	0.107	0.265	0.027	0.087	0.045	0.12	-0.416	0.154	0.123	-0.396	-0.387	-0.337	0.188
	GC-Skew	-0.281	-0.5	-0.075	-0.139	-0.127	-0.235	0.303	-0.229	-0.333	0.302	0.396	0.272	-0.373

Lopaphus sphalerus	length	675	159	1534	670	787	1134	966	1020	352	1332	291	1723	480
	A+T%	76.9	88.1	71.4	72.8	73.6	73.8	77.9	82.1	77.2	78.7	81.8	79.7	82.4
	AT-Skew	0.11	0.214	0.048	0.184	0.054	0.111	-0.431	0.123	0.14	-0.407	-0.487	-0.353	0.121
	GC-Skew	-0.179	-0.368	-0.032	-0.143	-0.087	-0.152	0.262	-0.169	-0.3	0.279	0.358	0.211	-0.262
Phraortes lianzhouensis	length	675	159	1534	667	787	1131	964	993	354	1326	285	1724	471
	A+T%	77.5	85.5	69.7	74.2	72.5	73	78.1	83.2	77.7	78.3	82.1	78.4	84.3
	AT-Skew	0.117	0.265	-0.007	0.131	0.007	0.056	-0.402	0.133	0.047	-0.372	-0.385	-0.339	0.139
	GC-Skew	-0.184	-0.304	-0.045	-0.093	-0.023	-0.102	0.249	-0.102	-0.215	0.174	0.412	0.204	-0.378
Marmessoidea bispinus	length	669	159	1534	670	789	1137	966	999	352	1332	291	1713	483
	A+T%	75.7	81.1	67.9	70.3	69.2	71.3	76.7	78.9	76.4	77.2	79.4	77.3	82.2
	AT-Skew	0.091	0.225	0.051	0.151	0.07	0.091	-0.406	0.153	0.063	-0.366	-0.368	-0.311	0.123
	GC-Skew	-0.288	-0.133	-0.077	-0.146	-0.086	-0.174	0.2	-0.171	-0.277	0.171	0.4	0.285	-0.326
Lopaphus albopunctatus	length	675	159	1534	670	789	1134	966	1014	352	1332	285	1725	474
	A+T%	78.7	90.5	73.6	75.8	74.4	75.7	80.7	84	80.7	80.9	82.5	81	84.2
	AT-Skew	0.096	0.167	0.011	0.126	0.036	0.026	-0.346	0.146	0.063	-0.348	-0.387	-0.296	0.103
	GC-Skew	-0.069	-0.333	0.022	-0.16	0	-0.043	0.161	-0.099	-0.235	0.2	0.120	0.152	-0.307
