# Supplementary material for: Nine Mitochondrial Genomes of Phasmatodea with Two Novel Mitochondrial Gene Rearrangements and Phylogeny
Source: Insects. 2023 May 22;14(5):485. doi: 10.3390/insects14050485 (PMC10231101; doi:10.3390/insects14050485)
Supplement: Supplementary file 1 [file insects-14-00485-s001.zip › TableS6.pdf]

Table S6. Codon usage of the 13 PCGs of the newly sequenced mitogenomes.

	Sungaya inexpectata		*Pulchriphyllium giganteum*		*pulchriphyllium bioculatum*		*Carausius* sp.		*Stheneboea repudiosa*		*Lopaphus sphalerus*		*Phraortes lianzhouensis*		*Marmessoidea bispinus*		*Lopaphus albopunctatus*	
	Count	RSCU	Count	RSCU	Count	RSCU	Count	RSCU	Count	RSCU	Count	RSCU	Count	RSCU	Count	RSCU	Count	RSCU
UUU(F)	293	1.61	307	1.66	299	1.66	253	1.63	296	1.71	327	1.77	300	1.73	264	1.6	320	1.84
UUC(F)	70	0.39	62	0.34	61	0.34	58	0.37	50	0.29	43	0.23	47	0.27	66	0.4	28	0.16
UUA(L)	270	3.58	264	3.36	267	3.58	338	4.13	357	4.42	316	4.19	346	4.42	326	4.01	407	5.02
UUG(L)	53	0.7	63	0.8	59	0.79	45	0.55	45	0.56	57	0.75	39	0.5	37	0.45	17	0.21
CUU(L)	47	0.62	56	0.71	44	0.59	42	0.51	24	0.3	31	0.41	35	0.45	42	0.52	30	0.37
CUC(L)	10	0.13	5	0.06	5	0.07	2	0.02	2	0.02	1	0.01	0	0	4	0.05	0	0
CUA(L)	69	0.91	74	0.94	69	0.92	59	0.72	56	0.69	45	0.6	48	0.61	77	0.95	32	0.4
CUG(L)	4	0.05	9	0.11	4	0.05	5	0.06	1	0.01	3	0.04	2	0.03	2	0.02	0	0
AUU(I)	285	1.61	255	1.57	299	1.69	340	1.76	341	1.74	331	1.8	337	1.78	283	1.62	350	1.85
AUC(I)	70	0.39	69	0.43	55	0.31	47	0.24	52	0.26	37	0.2	42	0.22	66	0.38	28	0.15
AUA(M)	332	1.79	336	1.71	362	1.81	358	1.79	373	1.9	396	1.87	349	1.86	359	1.81	384	1.91
AUG(M)	39	0.21	57	0.29	39	0.19	42	0.21	20	0.1	27	0.13	27	0.14	37	0.19	19	0.09
GUU(V)	87	1.82	102	2.16	86	2.06	56	1.44	70	1.81	70	1.79	81	1.78	77	1.69	61	1.56
GUC(V)	3	0.06	7	0.15	8	0.19	5	0.13	2	0.05	4	0.1	5	0.11	10	0.22	2	0.05
GUA(V)	91	1.91	71	1.5	67	1.6	86	2.21	77	1.99	78	2	90	1.98	83	1.82	89	2.28
GUG(V)	10	0.21	9	0.19	6	0.14	9	0.23	6	0.15	4	0.1	6	0.13	12	0.26	4	0.1
UCU(S)	95	2.28	88	1.94	92	2.05	89	2.18	100	2.31	100	2.33	96	2.3	101	2.36	99	2.45
UCC(S)	18	0.43	18	0.4	15	0.33	13	0.32	9	0.21	12	0.28	6	0.14	5	0.12	7	0.17
UCA(S)	108	2.59	127	2.81	121	2.7	118	2.89	106	2.45	115	2.67	117	2.8	101	2.36	115	2.85
UCG(S)	4	0.1	4	0.09	5	0.11	3	0.07	3	0.07	1	0.02	4	0.1	5	0.12	0	0
CCU(P)	53	1.61	40	1.21	47	1.4	67	2.03	52	1.6	49	1.5	57	1.68	57	1.77	56	1.68
CCC(P)	16	0.48	18	0.55	14	0.42	10	0.3	7	0.22	11	0.34	10	0.29	12	0.37	4	0.12
CCA(P)	59	1.79	70	2.12	70	2.09	53	1.61	68	2.09	70	2.14	68	2	58	1.8	72	2.17
CCG(P)	4	0.12	4	0.12	3	0.09	2	0.06	3	0.09	1	0.03	1	0.03	2	0.06	1	0.03
ACU(T)	52	0.91	57	1.07	49	0.92	70	1.47	67	1.31	64	1.32	53	1.14	67	1.34	63	1.32
ACC(T)	29	0.51	23	0.43	33	0.62	20	0.42	16	0.31	15	0.31	15	0.32	18	0.36	3	0.06
ACA(T)	142	2.49	128	2.39	127	2.37	99	2.07	122	2.38	112	2.31	113	2.43	109	2.18	125	2.62
ACG(T)	5	0.09	6	0.11	5	0.09	2	0.04	0	0	3	0.06	5	0.11	6	0.12	0	0
GCU(A)	44	1.54	38	1.49	39	1.58	49	1.75	44	1.69	41	1.41	53	1.83	53	1.67	46	1.7

GCC(A)	13	0.46	12	0.47	15	0.61	11	0.39	4	0.15	8	0.28	11	0.38	13	0.41	4	0.15
GCA(A)	52	1.82	49	1.92	43	1.74	50	1.79	56	2.15	66	2.28	50	1.72	56	1.76	57	2.11
GCG(A)	5	0.18	3	0.12	2	0.08	2	0.07	0	0	1	0.03	2	0.07	5	0.16	1	0.04
UAU(Y)	140	1.64	126	1.57	148	1.68	167	1.87	155	1.79	150	1.8	165	1.83	151	1.64	170	1.9
UAC(Y)	31	0.36	34	0.42	28	0.32	12	0.13	18	0.21	17	0.2	15	0.17	33	0.36	9	0.1
CAU(H)	42	1.27	36	1.16	40	1.27	53	1.54	43	1.39	47	1.45	50	1.54	44	1.28	59	1.79
CAC(H)	24	0.73	26	0.84	23	0.73	16	0.46	19	0.61	18	0.55	15	0.46	25	0.72	7	0.21
CAA(Q)	54	1.66	55	1.64	50	1.59	53	1.74	58	1.9	56	1.75	57	1.78	51	1.73	57	1.9
CAG(Q)	11	0.34	12	0.36	13	0.41	8	0.26	3	0.1	8	0.25	7	0.22	8	0.27	3	0.1
AAU(N)	141	1.51	134	1.43	138	1.45	175	1.76	173	1.75	192	1.81	158	1.71	141	1.56	195	1.84
AAC(N)	46	0.49	53	0.57	52	0.55	24	0.24	25	0.25	20	0.19	27	0.29	40	0.44	17	0.16
AAA(K)	86	1.7	96	1.64	103	1.72	104	1.79	98	1.78	95	1.76	108	1.79	98	1.73	104	1.82
AAG(K)	15	0.3	21	0.36	17	0.28	12	0.21	12	0.22	13	0.24	13	0.21	15	0.27	10	0.18
GAU(D)	54	1.54	54	1.61	51	1.55	65	1.71	60	1.67	54	1.61	65	1.76	58	1.59	67	1.86
GAC(D)	16	0.46	13	0.39	15	0.45	11	0.29	12	0.33	13	0.39	9	0.24	15	0.41	5	0.14
GAA(E)	69	1.73	57	1.61	58	1.66	62	1.85	64	1.75	67	1.74	66	1.78	54	1.4	75	1.92
GAG(E)	11	0.28	14	0.39	12	0.34	5	0.15	9	0.25	10	0.26	8	0.22	23	0.6	3	0.08
UGU(C)	40	1.82	45	1.73	53	1.86	39	1.86	41	1.95	43	1.91	44	1.83	41	1.78	42	1.95
UGC(C)	4	0.18	7	0.27	4	0.14	3	0.14	1	0.05	2	0.09	4	0.17	5	0.22	1	0.05
UGA(W)	82	1.71	71	1.54	75	1.63	89	1.82	81	1.78	83	1.77	83	1.78	79	1.66	90	1.91
UGG(W)	14	0.29	21	0.46	17	0.37	9	0.18	10	0.22	11	0.23	10	0.22	16	0.34	4	0.09
CGU(R)	14	1.06	14	1.14	13	1.06	19	1.46	19	1.49	20	1.54	20	1.54	15	1.13	15	1.13
CGC(R)	0	0	0	0	2	0.16	2	0.15	1	0.08	1	0.08	3	0.23	2	0.15	0	0
CGA(R)	32	2.42	30	2.45	30	2.45	26	2	27	2.12	30	2.31	29	2.23	30	2.26	36	2.72
CGG(R)	7	0.53	5	0.41	4	0.33	5	0.38	4	0.31	1	0.08	0	0	6	0.45	2	0.15
AGU(S)	29	0.69	33	0.73	34	0.76	30	0.73	34	0.79	39	0.91	39	0.93	38	0.89	28	0.69
AGC(S)	3	0.07	4	0.09	5	0.11	4	0.1	4	0.09	1	0.02	1	0.02	7	0.16	3	0.07
AGA(S)	71	1.7	82	1.81	79	1.76	65	1.59	82	1.9	70	1.63	69	1.65	84	1.96	69	1.71
AGG(S)	6	0.14	6	0.13	8	0.18	5	0.12	8	0.18	6	0.14	2	0.05	1	0.02	2	0.05
GGU(G)	73	1.4	72	1.43	67	1.38	75	1.53	83	1.76	76	1.58	82	1.64	57	1.19	87	1.76
GGC(G)	14	0.27	11	0.22	9	0.19	4	0.08	4	0.08	4	0.08	2	0.04	8	0.17	1	0.02
GGA(G)	74	1.42	85	1.69	78	1.61	97	1.98	86	1.82	87	1.8	90	1.8	92	1.92	93	1.88
GGG(G)	48	0.92	33	0.66	40	0.82	20	0.41	16	0.34	26	0.54	26	0.52	35	0.73	17	0.34
