# Supplementary material for: Nine Mitochondrial Genomes of Phasmatodea with Two Novel Mitochondrial Gene Rearrangements and Phylogeny
Source: Insects. 2023 May 22;14(5):485. doi: 10.3390/insects14050485 (PMC10231101; doi:10.3390/insects14050485)
Supplement: Supplementary file 1 [file insects-14-00485-s001.zip › TableS7.pdf]

Table S7. Amino acid use frequencies of proteins in the newly sequenced mitogenomes.

| species | *Sungaya inexpectata* | | *Pulchriphyllium bioculatum* | | *Pulchriphyllium giganteum* | | *Carausius* sp. | | *Stheneboea repudiosa* | | *Lopaphus sphalerus* | | *Phraortes lianzhouensis* | | *Marmessoidea bispinus* | | *Lopaphus albopunctatus* | |
|---|---|---|---|---|---|---|---|---|---|---|---|---|---|---|---|---|---|---|
| AA | Count | % | Count | % | Count | % | Count | % | Count | % | Count | % | Count | % | Count | % | Count | % |
| Phe(F) | 363 | 9.86 | 360 | 9.79 | 369 | 10.02 | 311 | 8.49 | 346 | 9.4 | 370 | 10 | 347 | 9.42 | 330 | 8.96 | 348 | 9.42 |
| Leu2(L2) | 323 | 8.77 | 326 | 8.87 | 327 | 8.88 | 383 | 10.46 | 402 | 10.93 | 373 | 10.08 | 385 | 10.46 | 363 | 9.85 | 424 | 11.47 |
| Leu1(L1) | 130 | 3.53 | 122 | 3.32 | 144 | 3.91 | 108 | 2.95 | 83 | 2.26 | 80 | 2.16 | 85 | 2.31 | 125 | 3.39 | 62 | 1.68 |
| Ile(I) | 355 | 9.64 | 354 | 9.63 | 324 | 8.8 | 387 | 10.57 | 393 | 10.68 | 368 | 9.95 | 379 | 10.29 | 349 | 9.47 | 378 | 10.23 |
| Met(M) | 371 | 10.07 | 401 | 10.91 | 393 | 10.68 | 400 | 10.92 | 393 | 10.68 | 423 | 11.44 | 376 | 10.21 | 396 | 10.75 | 403 | 10.91 |
| Val(V) | 191 | 5.19 | 167 | 4.54 | 189 | 5.13 | 156 | 4.26 | 155 | 4.21 | 156 | 4.22 | 182 | 4.94 | 182 | 4.94 | 156 | 4.22 |
| Ser2(S2) | 225 | 6.11 | 233 | 6.34 | 237 | 6.44 | 223 | 6.09 | 218 | 5.93 | 228 | 6.16 | 223 | 6.06 | 212 | 5.75 | 221 | 5.98 |
| Pro(P) | 132 | 3.58 | 134 | 3.65 | 132 | 3.59 | 132 | 3.6 | 130 | 3.53 | 131 | 3.54 | 136 | 3.69 | 129 | 3.5 | 133 | 3.6 |
| Thr(T) | 228 | 6.19 | 214 | 5.82 | 214 | 5.81 | 191 | 5.22 | 205 | 5.57 | 194 | 5.24 | 186 | 5.05 | 200 | 5.43 | 191 | 5.17 |
| Ala(A) | 114 | 3.1 | 99 | 2.69 | 102 | 2.77 | 112 | 3.06 | 104 | 2.83 | 116 | 3.14 | 116 | 3.15 | 127 | 3.45 | 108 | 2.92 |
| Tyr(Y) | 171 | 4.64 | 176 | 4.79 | 160 | 4.35 | 179 | 4.89 | 173 | 4.7 | 167 | 4.51 | 180 | 4.89 | 184 | 4.99 | 179 | 4.84 |
| His(H) | 66 | 1.79 | 63 | 1.71 | 62 | 1.68 | 69 | 1.88 | 62 | 1.69 | 65 | 1.76 | 65 | 1.77 | 69 | 1.87 | 66 | 1.79 |
| Gln(Q) | 65 | 1.76 | 63 | 1.71 | 67 | 1.82 | 61 | 1.67 | 61 | 1.66 | 64 | 1.73 | 64 | 1.74 | 59 | 1.6 | 60 | 1.62 |
| Asn(N) | 187 | 5.08 | 190 | 5.17 | 187 | 5.08 | 199 | 5.43 | 198 | 5.38 | 212 | 5.73 | 185 | 5.02 | 181 | 4.91 | 212 | 5.74 |
| Lys(K) | 101 | 2.74 | 120 | 3.26 | 117 | 3.18 | 116 | 3.17 | 110 | 2.99 | 108 | 2.92 | 121 | 3.29 | 113 | 3.07 | 114 | 3.09 |
| Asp(D) | 70 | 1.9 | 66 | 1.8 | 67 | 1.82 | 76 | 2.08 | 72 | 1.96 | 67 | 1.81 | 74 | 2.01 | 73 | 1.98 | 72 | 1.95 |
| Glu(E) | 80 | 2.17 | 70 | 1.9 | 71 | 1.93 | 67 | 1.83 | 73 | 1.98 | 77 | 2.08 | 74 | 2.01 | 77 | 2.09 | 78 | 2.11 |
| Cys(C) | 44 | 1.19 | 57 | 1.55 | 52 | 1.41 | 42 | 1.15 | 42 | 1.14 | 45 | 1.22 | 48 | 1.3 | 46 | 1.25 | 43 | 1.16 |
| Trp(W) | 96 | 2.61 | 92 | 2.5 | 92 | 2.5 | 98 | 2.68 | 91 | 2.47 | 94 | 2.54 | 93 | 2.53 | 95 | 2.58 | 94 | 2.54 |
| Arg(R) | 53 | 1.44 | 49 | 1.33 | 49 | 1.33 | 52 | 1.42 | 51 | 1.39 | 52 | 1.41 | 52 | 1.41 | 53 | 1.44 | 53 | 1.43 |
| Ser1(S1) | 109 | 2.96 | 126 | 3.43 | 125 | 3.4 | 104 | 2.84 | 128 | 3.48 | 116 | 3.14 | 111 | 3.01 | 130 | 3.53 | 102 | 2.76 |
| Gly(G) | 209 | 5.67 | 194 | 5.28 | 201 | 5.46 | 196 | 5.35 | 189 | 5.14 | 193 | 5.22 | 200 | 5.43 | 192 | 5.21 | 198 | 5.36 |
