# Supplementary material for: Nine Mitochondrial Genomes of Phasmatodea with Two Novel Mitochondrial Gene Rearrangements and Phylogeny
Source: Insects. 2023 May 22;14(5):485. doi: 10.3390/insects14050485 (PMC10231101; doi:10.3390/insects14050485)
Supplement: Supplementary file 1 [file insects-14-00485-s001.zip › TableS8.pdf]

Table S8. Start and stop codons of the 13 PCGs in the sequenced mitochondrial genes.

Gene \ Specise	*Sungaya inexpectata*	*Pulchriphyllium bioculatum*	*Pulchriphyllium giganteum*	*Carausius* sp	*Stheneboea repudiosa*	*Lopaphus sphalerus*	*Phraortes lianzhouensis*	*Marmessoidea bispinus*	*Lopaphus albopunctatus*
ATP6	ATA/TAA	ATA/TAA	ATA/TAA	ATA/TAA	ATA/TAA	ATA/TAA	ATA/TAA	ATA/TAA	ATA/TAA
ATP8	ATT/TAA	ATA/TAA	ATA/TAA	ATA/TAA	ATA/TAA	ATT/TAA	ATG/TAA	ATG/TAA	ATA/TAA
COX1	ATG/T	ATG/T	ATG/T	ATG/T	ATG/T	ATG/T	ATG/T	ATG/T	ATG/T
COX2	ATA/T	ATT/T	ATA/T	ATA/T	ATA/TAA	ATA/T	ATA/T	ATA/T	ATA/T
COX3	ATG/TAA	ATG/TAA	ATG/TAA	ATG/TAA	ATG/TAA	ATG/T	ATG/T	ATG/TAA	ATG/TAA
Cytb	ATG/TAG	ATG/TAA	ATG/TAA	ATG/TAA	ATG/TAG	ATG/TAA	ATG/TAG	ATG/TAA	ATG/TAG
ND1	ATT/TAA	ATA/TAA	ATA/TAG	TTG/TAA	TTG/TAA	ATA/TAA	ATT/T	ATA/TAA	ATA/TAA
ND2	ATT/TAA	ATT/TAA	ATA/TAA	ATC/TAA	ATA/TAA	ATT/TAA	ATC/TAA	ATT/TAA	ATT/TAA
ND3	ATT/TAG	ATA/T	ATA/T	ATT/TAA	ATT/TAG	ATA/T	ATT/TAG	ATT/T	ATT/T
ND4	ATG/TAA	ATG/T	ATG/T	ATG/TAG	ATG/T	ATG/TAA	ATA/TAG	GTG/TAG	ATG/TAA
ND4L	ATT/TAA	ATT/TAA	ATG/TAA	ATA/TAA	ATA/TAA	ATT/TAA	ATA/TAA	ATG/TAA	ATT/TAA
ND5	ATT/TA	ATA/TAG	ATT/TAA	ATT/TA	ATT/T	ATT/T	ATT/TA	ATT/TAA	ATT/TAA
ND6	ATT/TAA	ATT/TAA	ATA/TAA	ATA/TAA	ATA/TAA	ATATAA	ATA/TAA	ATT/TAA	ATA/TAA
